# MiProChip: A Scalable Microfluidic Platform for Multiplexed Single-Cell Proteomics via Isobaric Labeling

**DOI:** 10.1021/acs.analchem.5c07275

**Published:** 2026-02-25

**Authors:** Tsai-Fang Chou, Huan-Chi Chiu, Sofani Tafesse Gebreyesus, Guan-Fu Chen, Yi-Ju Chen, Abigail Ruth F. Velasquez, Kuo-I Lin, Yu-Ju Chen, Hsiung-Lin Tu

**Affiliations:** † Institute of Chemistry, 71552 Academia Sinica, Taipei 11529, Taiwan; ‡ Department of Chemistry, National Taiwan University, Taipei 10617, Taiwan; § Genomics Research Center, Academia Sinica, Taipei 115, Taiwan; ∥ Chemical Biology and Molecular Biophysics, Taiwan International Graduate Program, Academia Sinica, Taipei 115, Taiwan; ⊥ Graduate Institute of Biochemical Sciences, National Taiwan University, Taipei 110, Taiwan; # Genome and Systems Biology Degree Program, Academia Sinica and National Taiwan University, Taipei 110, Taiwan

## Abstract

Single-cell proteomics (SCP) platforms are widely sought-after biomedical tools to complement existing omics technologies. Here, we present MiProChip, a microfluidic platform that integrates cell capture, lysis, protein digestion, tandem mass tag (TMT) labeling, on-chip pooling, and desalting a streamlined workflow for multiplexed SCP profiling. We optimized a chip-compatible TMT multiplexing protocol with a carrier-boosting strategy, enabling high-throughput operation and deep proteome coverage. MiProChip was designed to effectively reduce the mass spectrometry (MS) operation time, minimize adsorptive losses, enhance mixing, and stabilize flow for on-chip pooling, leading to a superior performance in recovery. Using PC9 and H1975 cells with a 100-cell carrier, a total of 3362 protein groups with 2775 ± 36 proteins were confidently identified across TMT-10-plex single-cell channels. Demonstration on murine colon adenocarcinoma cells identified 3199 proteins with 1669 ± 261 proteins per single cell to characterize galectin-8- and TGF-β-specific responses. Single-cell principal component analysis (PCA) showed separation of the control from treated groups, partial overlap between galectin-8 and TGF-β, and close clustering of TGF-β with the combination treatment, supporting a dominant TGF-β effect. Pathway enrichment analysis reveals their responsive pathway and distinguishes galectin-8- and TGF-β-specific responses, revealing downregulation of metastasis-related markers to support antimetastasis potential of galectin-8, which was not detected by bulk proteomic analysis. Collectively, MiProChip captured subtle proteomic heterogeneity and treatment-dependent single-cell responses, establishing a sensitive and robust platform for high-throughput SCP analysis.

## Graphical abstract

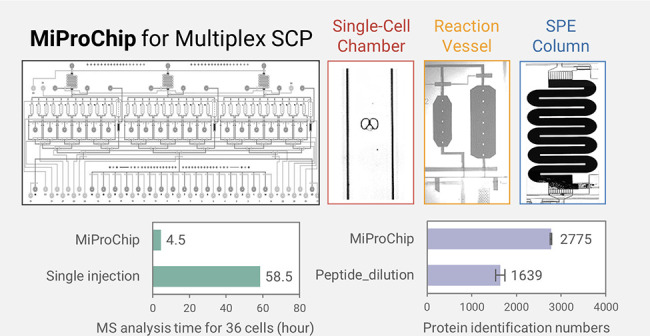

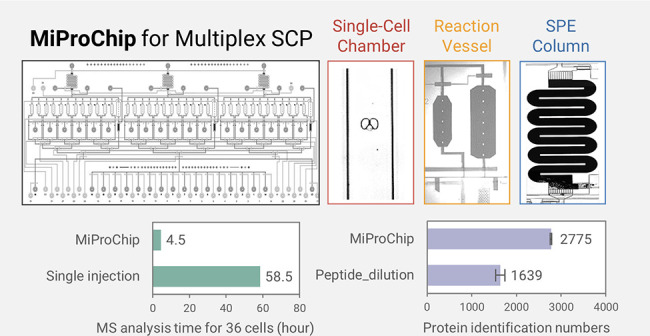

## Introduction

Mass spectrometry (MS)-based proteomics has advanced rapidly in both instrumentation and data analysis, enabling the quantification of thousands of proteins for various biological samples. While conventional MS approaches allow comparison of proteomic profiles across bulk samples (10^4^–10^6^ cells), they often obscure differences between individual cell types and fail to resolve cell-to-cell variability. Single-cell proteomics (SCP), by contrast, holds the key to deciphering cellular heterogeneity, mapping intercellular regulatory networks, and understanding disease progression.^1–3^ Unlike genomics and transcriptomics—which have been revolutionized by droplet-based sequencing^4–8^ and being implemented broadly in single-cell research—the development of reliable platforms for high-throughput SCP analysis remains an active research area with new developments underway.^9–11^ This limitation primarily arises from the extremely low protein content of single cells and the technical challenges of handling such small samples. Compared to conventional bulk sample processing, reducing this sample input down to the single-cell level introduces challenges associated with extremely low protein amounts per cell and unwanted protein/peptide losses during small-volume sample handling.

To address the limitation, several chip-based strategies have been developed to show improved proteome identification from limited cell samples.^12–17^ Meanwhile, microfluidic platforms such as the nanoPOTS^18^ and SciProChip^19^ were employed to confine reactions in nanoliter volumes and integrate multiple steps to achieve SCP analysis. Furthermore, higher-throughput analysis for multiplexed SCP presents another unmet need for the simultaneous profiling of single cells. Throughput and proteome coverage can be improved with carrier-based strategies, such as tandem mass tag (TMT) isobaric labeling as reported in the SCP assays including SCoPE-MS,^20^ SCoPE2,^21^ and nanoPOTS.^22^ In these systems, single-cell samples are processed and isotopically labeled individually and multiplexed together with a high-abundance “carrier” channel to boost peptide identifications. These assays demonstrated key technical advances, including the first TMT-based single-cell workflow in SCoPE-MS, improved sensitivity and protein coverage in SCoPE2, and high-efficiency processing of single cells in nanoPOTS. Collectively, they showcased enhanced throughput and analytical depth. More broadly, these studies demonstrate that isobaric labeling could enhance 10- to-16-fold multiplexing for SCP, enabling efficient data acquisition and increasing the experimental throughput.

In this study, leveraging our previous work on SciProChip for SCP analysis, we present a new microfluidic platform that incorporates TMT labeling to advance the overall throughput. While polydimethylsiloxane (PDMS) systems are broadly used in microfluidic devices because of their flexibility, ease, and low cost in fabrication, the integration of TMT chemistry requires overcoming reagent incompatibility, limited reaction volume, and incomplete labeling that can compromise quantitative accuracy. Particularly, multichannel isotopic labeling of picogram-level samples in the confined microenvironment demands precise fluidic handling and reaction optimization to achieve complete multiplexed labeling. MiProChip developed herein overcomes these barriers and enables carrier-assisted pooling of up to 12 single cells per MS run, achieving more than a 10-fold reduction in instrument time while maintaining high proteome coverage (1945–2761 protein groups per run in all samples). MiProChip enables efficient on-chip processing, from proteomic workflow (cell capture, cell lysis, protein digestion, and peptide cleanup) to isotopic labeling (peptide labeling, carrier incorporation, and channel pooling), eliminating single-cell-by-single-cell analysis and greatly reducing MS acquisition time. The feature of the on-chip pooling design minimizes off-chip pooling steps prior to liquid chromatography (LC) injection. Pooling/transfer steps may involve additional manual handling or centrifugation-assisted steps that introduce sample loss and environmental exposure.^23^ This integrated design lowers surface-to-volume exposure and helps reduce the potential adsorption and contamination for low-input samples. Together, these advances establish MiProChip as a reproducible and scalable workflow for streamlined, high-throughput SCP (Scheme 1). The feasibility and performance of MiProChip were evaluated by non-small cell lung cancer PC9 and H1975 single cells. The results showed that the proposed workflow was robust and showcased consistent performance across biological replicates. Furthermore, its general applicability was demonstrated by quantitative profiling of galectin-8-mediated regulation in murine colon adenocarcinoma MC38 cells and their individual cell responses. Collectively, these results demonstrated the analytical performance and applicability to delineate the treatment-dependent cellular heterogeneity masked in bulk sample analyses.

**Scheme 1:**
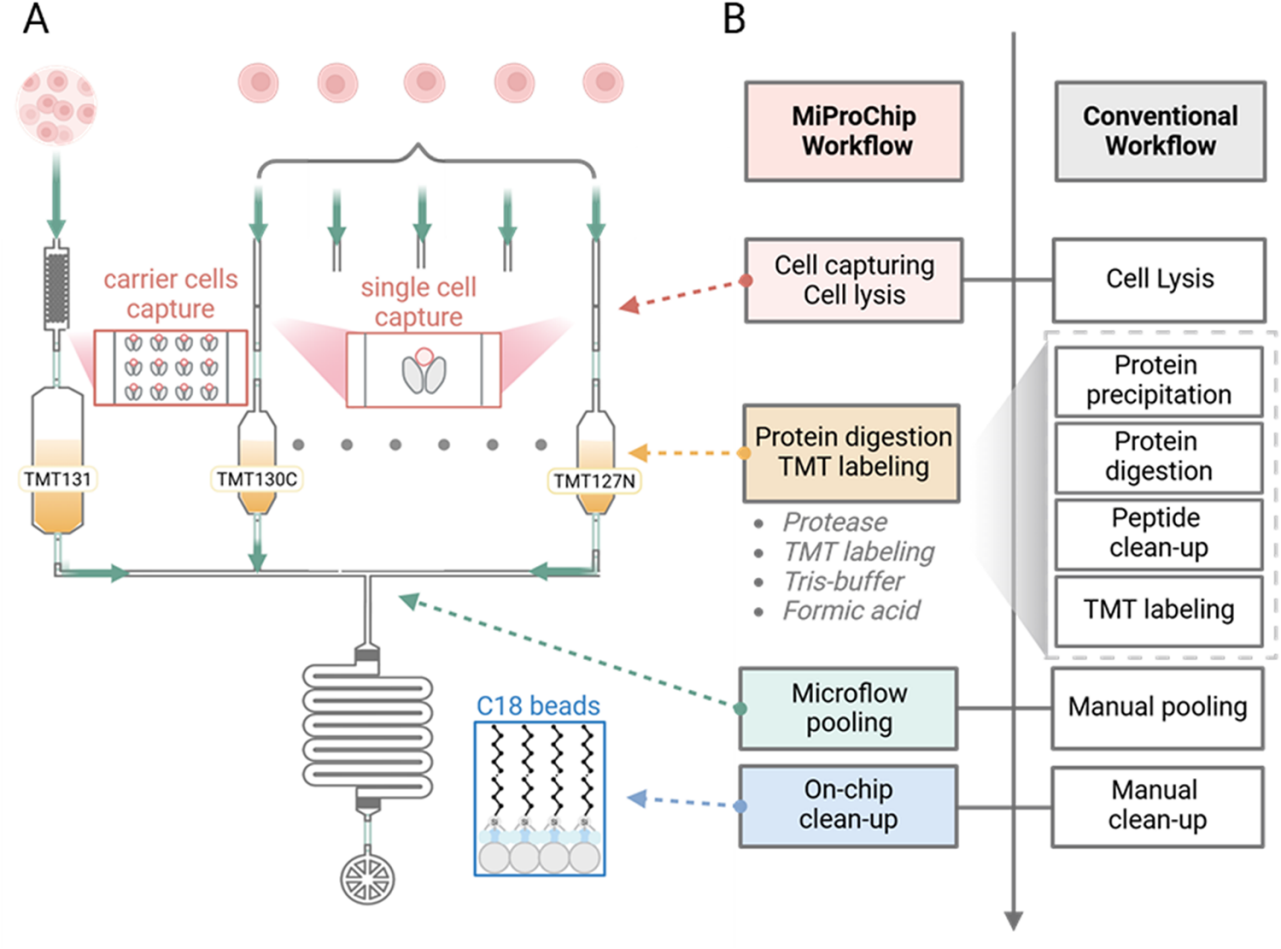
Design of MiProChip for TMT-Based Proteomics Workflow^a^

a (A) Schematic diagram of MiProChip, showing the design of the carrier channel (TMT-131) for boosting signal intensities and sampling channels using TMT-10-plex tag and (B) the whole proteomic processing features an automated and simplified workflow compared to the conventional TMT labeling workflow.

## Experimental Section

### Materials and Reagents

RTV 615-J polydimethylsiloxane (PDMS) prepolymer and curing agent were purchased from Momentive Performance Materials (MO). SU-8 3025 negative photoresist was purchased from Nippon Kayaku (Tokyo, Japan), and AZ 40XT-11D positive photoresist was purchased from Merck KGaA (Darmstadt, Germany) and used for mold fabrication. Triethylammonium bicarbonate (TEAB), tris­(2-carboxyethyl)­phosphine (TCEP), chloroacetamide (CAA), chlorotrimethylsilane, *N*-dodecyl-β-d-maltoside (DDM), sodium laurate (SL), protease inhibitor cocktail set III (EDTA-free-Calbiochem), and trifluoroacetic acid (TFA) were purchased from Sigma-Aldrich (MO). Formic acid (FA) was purchased from Honeywell Fluka (NC) and freshly prepared in ddH_2_O before use. LC-MS-grade acetonitrile (ACN), TMT labeling reagents (TMT-10 kit), and a Pierce BCA protein assay kit were purchased from Thermo Fisher Scientific (MA). RapiGest SF surfactant was purchased from Waters (MA) and dissolved in a fresh 50 mM triethylammonium bicarbonate buffer with a concentration of 0.3% (w/v), aliquoted, and stored at −30 °C until further use. Lys-C (MS-grade) and trypsin (MS-grade) were purchased from Promega (WI). VDSpher C18 beads (5 μm; 300 Å pore size) were purchased from VDS Optilab Chromatographietechnik GmbH (Berlin, Germany).

### Cell Culture

Human lung adenocarcinoma cell lines, PC9 and H1975, were purchased from ATCC (VA) and cultured at 37 °C with 5% CO_2_ in RPMI-1640 medium supplemented with 0.375% (w/v) N-(2-hydroxyethyl)­piperazine-N′-2-ethanesulfonic acid (HEPES), 0.22% (w/v) sodium bicarbonate, 0.01% (w/v) sodium pyruvate, 10% (v/v) fetal bovine serum (FBS), and 1% (v/v) penicillin–streptomycin–amphotericin.

Murine colon adenocarcinoma cell line (MC38) was purchased from Kerafast, Inc. (Cat. No. ENH204-FP) and grown in Dulbecco’s modified Eagle’s medium (DMEM) supplemented with 1% (v/v) nonessential amino acid, 1% (v/v) sodium pyruvate, 1% (v/v) GlutaMAX (Cat. No. 35050061, Gibco), 1% (v/v) HEPES, 10% (v/v) FBS, and 1% (v/v) penicillin (100 U/mL)/streptomycin (100 μg/mL) at 37 °C with 5% CO_2_. The cell lines were maintained to have approximately 80–95% confluency. Before treatment, the MC38 cells were seeded in a 6-well plate at a cell number of 2 × 10^5^ cells per well in 2 mL of media and incubated for 24 h at 37 °C under 5% CO_2_ atmosphere. After 24 h, the spent media was replaced with 2 mL of media containing the following treatments: 2.5 μM recombinant human galectin-8 (TPG Biologics, Inc.), 10 ng/mL TGF-β (Cat. No. 763102, Biolegend), and combined treatment. For the control, 2 mL of media without treatment was transferred into the MC38 cells. The cells were incubated for 48 h at 37 °C with 5% CO_2_. Afterward, the treated cells were harvested at a cell density of approximately 1 × 10^6^ cells/mL for SCP analysis.

### Chip Fabrication

The chip was designed by the AutoCAD software, and the photomasks were printed at 5 ± 0.5 μm resolution by M&R Nano Technology Co. Ltd. (Taoyuan City, Taiwan) and Taiwan Kong King Co. Ltd. (Taoyuan City, Taiwan). Silicon wafer molds were fabricated using a mask aligner (EV-620) following the standard soft lithography protocol. The control layer was fabricated by spin-coating SU-8 3025 at 4200 rpm to yield 18 μm thickness. Meanwhile, the flow layer was composed of three layers, including a valve layer (24 μm, AZ 40XT-11D spin at 2700 rpm), a main flow layer (25 μm, SU-8 3025 spin at 3100 rpm), and an additional layer for reaction vessels (80 μm, SU-8 3025 spin at 1250 rpm).

For PDMS casting, 10 g of PDMS (10:1) was poured and spin-coated at 1800 rpm for the control layer using a spin coater (Laurell WS-650 HZ-23NPP/UD2 Spin coater). For the flow layer, 66 g of PDMS (10:1) was poured onto the flow layer mold and gently degassed in a desiccator. Both layers were baked at 80 °C in an oven. After curing, inlets and outlets were punched, surfaces were plasma-treated for 1 min, and then two layers were aligned and bonded by using a custom stereomicroscope. Following 80 °C curing, the assembled chip was punched for control valve inputs, followed by bonding to a plasma-pretreated glass slide, the chip was baked at 80 °C for 48 h and ready for subsequent use.

### Chip Characterization—Cell Capture, Mixing, and Flow Characterization

Non-small cell lung cancer PC9 cells with a density of 125 cells/μL were used to determine the cell capture efficiency. Before cell loading, the capture chambers were degassed and filled with phosphate-buffered saline (PBS). The cells were then introduced to the chambers through PEEK tubing at 3–6 psi. Bright-field microscopy was used to monitor and quantify capture events.

Mixing efficiency of the reaction vessels was examined by the color-dye mixing experiments and quantified by the relative mixing index (RMI) analysis, as reported in a previous study.^19^ Briefly, blue and yellow dyes were sequentially injected into reaction vessels of MiProChip, and the resulting mixing behavior was recorded by time-lapse microscopy and used to quantify the RMI. Additionally, a flow distribution characterization of reaction vessels for verifying the degree of preferential flow under either empty or packed solid-phase extraction (SPE) columns was performed by dye-based experiments. For such an experiment, all reaction vessels (i.e., both 1- and 100-cell units) were initially filled with yellow dye. Time-lapse microscopy was used to monitor the color change upon introduction of a colorless buffer into all chambers. Image analysis was applied to evaluate the preferential flow.

### TMT Experiments and Experimental Design

All SCP experiments reported employed a carrier-assisted, isobaric TMT labeling strategy.^20^ Individual single cells were isolated into dedicated TMT channels and combined with a carrier channel containing 100 cells, followed by subsequent LC-MS/MS analysis. Unless otherwise noted, each TMT experiment consisted of eight single-cell channels, one carrier channel, and one blank channel to mitigate isotopic leakage (Figure 4A). The carrier peptide samples were processed using the same on-chip workflow to ensure consistency in the proteomic workflow. TMT-10-plex reagents were used to demonstrate multiplexing in MiProChip (full channel layouts are provided in Table S1 and Figure 4A). For optimization of workflow and bulk sample experiment, including evaluations of acetonitrile percentage, surface passivation reagents, protease inhibitors, and MC38 bulk sample analysis, details are described in the Supporting Information.

For evaluation of MiProChip performance using PC9 and H1975 cells, the TMT labeling was designed as follows: channels 126–128N assigned to four PC9 single cells, channels 128C–130C assigned to four H1975 single cells, 130N reserved as a blank channel, and 131 used for a mixed 100-cell carrier (PC9:H1975, 1:1). For demonstration of MiProChip for biological experiment (galectin-8-mediated regulation in murine colon adenocarcinoma MC38 cells), a total of 40 single cells were analyzed across two MiProChips: untreated and galectin-8-treated cells on chip 1, and TGF-β-treated or dual-treated cells on chip 2. Carrier samples combining all four conditions (approximately 25 cells per condition) were labeled with TMT-131. Single-cell channels followed the same allocation pattern as above (126–128N for one condition group and 128C–130C for the other), with 130N intentionally left unused.

### Manual Processing for Bulk, Cell, and Peptide Diluted Sample

To compare with chip-based proteomic processing, samples were prepared to mimic single-cell input amounts using two approaches: (1) direct dilution of cells and (2) dilution of peptides.

#### Limiting Cell-Dilution to Single-Cell Equivalents

PC9 and H1975 cells were first trypsinized and collected at a concentration of 5 × 10^5^ cells/mL in a 10 mL tube, followed by centrifugation to remove the culture medium. The cell suspension was then serially diluted to concentrations corresponding to 50 cells/μL (carrier samples) and 0.5 cell/μL (single-cell samples). These samples were then aliquoted as 2 μL in PCR-8-strip tubes coated with 0.01% (w/v) DDM. Either 100 or 1 cell(s) were mixed with 5 μL of lysis buffer (containing 0.5% RapiGest, 50 mM CAA, and 10 mM TCEP in 50 mM TEAB), heated at 70 °C for 30 min, then 90 °C for 10 min, cooled to 37 °C, and subjected to 10 min sonication (BioRuptor). Samples were digested overnight using a Lys-C/Trypsin mix (0.6 μg for 100-cell samples; 0.006 μg for single-cell samples). Peptides were labeled with TMT-10-plex reagents same as the channel designed and quenched with 200 mM Tris buffer (pH = 8) to final concentration equal to 50 mM. Finally, samples labeled with different TMT channels are combined with the carrier samples, desalted using ZipTips, and prepared for LC-MS/MS analysis.

#### Peptide Dilution to Single-Cell Equivalents

Bulk PC9 and H1975 cells were lysed using sodium laurate (SL)-based lysis buffer (1% sodium laurate, 50 mM CAA, 10 mM TCEP in Tris buffer), and protein concentrations were determined via the bicinchoninic acid (BCA) assay. A total of 50 μg of protein from each cell line was digested overnight with Lys-C/Trypsin and desalted using SDB-XC-C18 StageTips. The desalted peptides were serially diluted to mimic 100-cell (20 ng) and 1-cell (0.2 ng) equivalents, followed by aliquoting to separate PCR-8-strip tubes coated with 0.01% DDM and underwent TMT labeling and quenching with 200 mM Tris buffer. As with cell-based dilutions, single-cell equivalents were combined with carrier samples, desalted by ZipTips, and prepared for LC-MS analysis.

### On-Chip Proteomic Processing

This study follows a previously reported protocol to perform the proteomics workflow using MiProChip.^19^ Briefly, the chip was connected via stainless steel connectors and Tygon tubing to the sinusoidal valves in a custom chip controller and mounted on an inverted microscope (Figure 1E). The custom controller was operated using MATLAB software. A working pressure of 25 psi was typically used to actuate the control valves. Prior to use, MiProChip channels were coated with 0.01% DDM for 1 h, rinsed with PBS, and dried under nitrogen. For bovine serum albumin (BSA) vs DDM comparisons, DDM was replaced with 0.01% (w/v) BSA. Reaction chambers were fully filled during coating (∼100 nL per chamber) and then rinsed with PBS to remove unbound BSA/DDM prior to drying. SPE columns were packed with C18 beads in an acetone suspension. Cells were lysed in microfluidic chambers using a RapiGest-based lysis buffer containing 0.5% (w/v) RapiGest, 50 mM chloroacetamide (CAA), and 10 mM tris­(2-carboxyethyl) phosphine (TCEP) in 50 mM TEAB, and the mixture was incubated at 70 °C for 30 min under shaking. For digestion optimization experiments only, a 1× protease inhibitor cocktail was included in the lysis buffer; protease inhibitors were omitted from the final optimized workflow. Digestion was performed with Lys-C and trypsin for 16 h at 40 °C. TMT reagents dissolved in 10% acetonitrile/100 mM TEAB were incubated for 1 h, followed by quenching with 20 nL 200 mM Tris buffer for another hour. Acidification was performed with 10% FA to reach a final concentration of 5% (v/v), and the reaction was incubated at 40 °C for 55 min. Before elution, SPE columns were preconditioned and equilibrated with desalting buffers (0, 50, 100% ACN in 0.1% TFA). The pooled peptides were desalted at 11 psi and collected directly from the MiProChip outlet using a pipet into a DDM-coated autosampler vial. The collected sample was dried in a SpeedVac and then reconstituted in 0.1% formic acid and loaded onto the Ultimate 3000 LC system for LC-MS/MS analysis.

**Figure 1:**
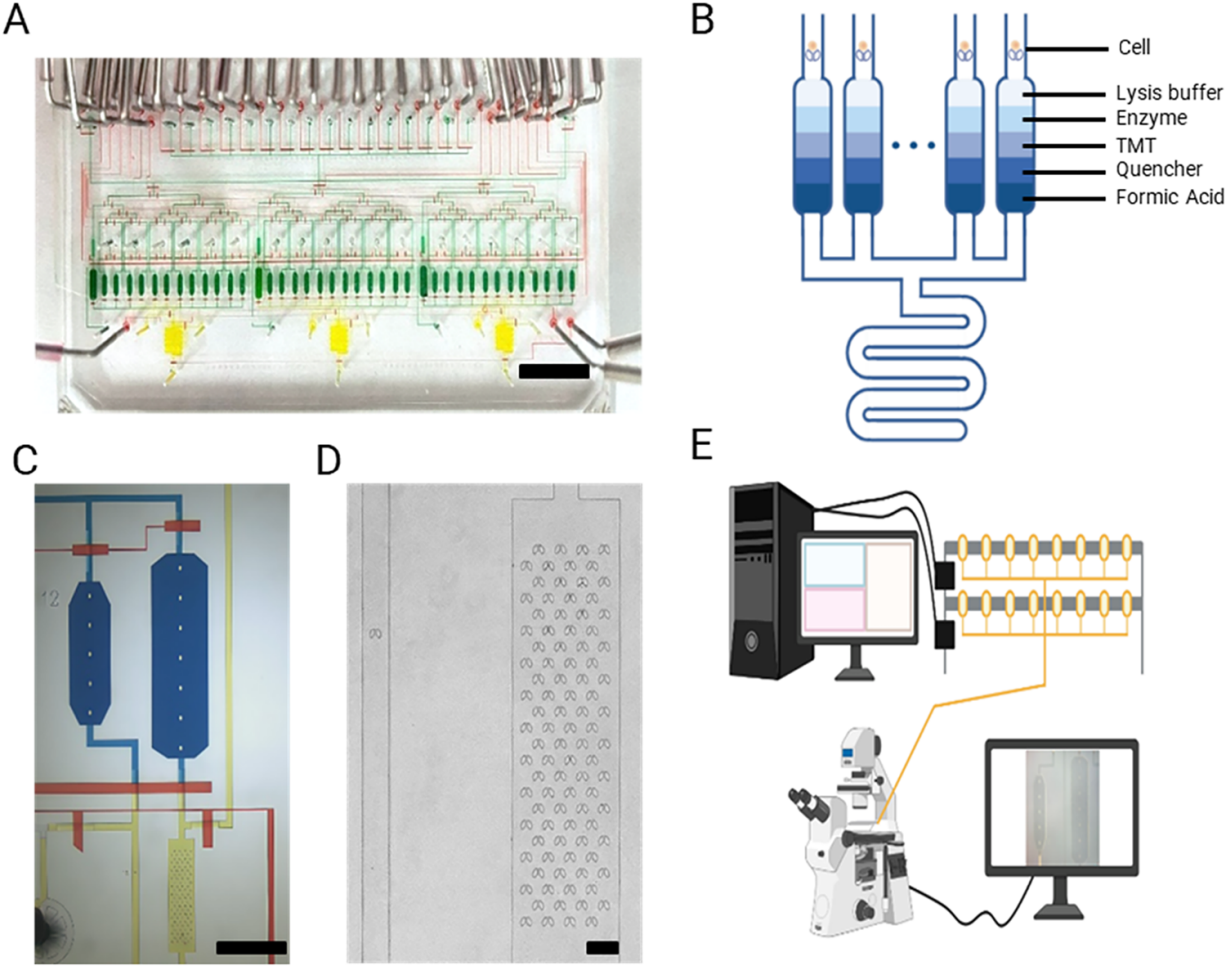
Schematic of the multiplexed isotopic labeling integrated proteomics chip (MiProChip) for TMT-based single-cell proteomics (SCP) workflow. (A) Bright-field image of MiProChip with functional modules stained with different dyes for visualization—cell capture chambers and reaction vessels in green, SPE columns in yellow, and control lines in red. Scale bar: 1 cm. (B) Illustration showing the sequential reagent additions in the reaction vessel during MiProChip workflow. (C) Close-up view showing a single-cell unit (left) and a 100-cell unit (right), featuring cell capture chambers (yellow) and reaction vessels (dark blue). Scale bar: 1 mm. (D) Bright-field image of a single-cell capture chamber (left) and a 100-cell capture chamber (right). Scale bar: 100 μm. (E) Schematic representation of the control system with MiProChip setup.

### LC-MS/MS Analysis

TMT-labeled single cells with carrier samples were analyzed on an Orbitrap Eclipse (Thermo Fisher Scientific) operated with Xcalibur software (version 4.3.73.11), coupled to an Ultimate 3000 RSLCnano system (Thermo Fisher Scientific). Desalted peptides were resuspended in 4.5 μL of loading buffer (0.1% FA) prior to analysis. Peptide separation was performed on a capillary C18 column (nanoEase, 130 Å, 1.7 μm, 75 μm × 250 mm; Waters) at a flow rate of 300 nL/min using buffer A (0.1% FA in water) and buffer B (0.1% FA in acetonitrile). The following 90 min gradient was applied: 2% B at 0 min; 6% B at 0.5 min; linear to 30% B at 52 min; 45% B at 63 min; 90% B at 74 min; hold at 90% B to 79 min; return to 1% B at 80 min; reequilibrate at 1% B to 90 min.

The mass spectrometer was operated in positive ion mode with a spray voltage of 1.75 kV, reimagined focus (RF) lens at 30%, and ion transfer tube temperature of 305 °C. Data-dependent acquisition (DDA) was performed with a cycle time of 3 s, selecting multiple charged precursors above an intensity threshold of 5 × 10^4^. Full MS scans were acquired at a resolution of 120,000, with an AGC target of 250% (normalized) and maximum injection time in auto mode, over a mass range of 400–1600 *m*/*z*. Precursors were isolated with a 0.7 *m*/*z* window (advanced peak determination enabled) and fragmented by higher-energy collisional dissociation (HCD) at a normalized collision energy (NCE) of 38%. MS/MS spectra were acquired in the Orbitrap at a resolution of 50,000, with an AGC target of 200% and maximum injection time of 86 ms. The details of workflow optimization and bulk sample experiments are presented in the Supporting Information (Supporting Methods).

### Protein Identification and Quantification

For protein identification, the raw files were processed in Proteome Discoverer (v3.1.1.93, Thermo Fisher Scientific) using the CHIMERYS algorithm^24^ (MSAID GmbH, Germany) in TMT DDA mode. Spectra were searched against the SWISS-PROT Human reference proteome^25^ (downloaded 2025-03-03; 20,340 sequences, 11,413,231 residues), supplemented with an in-house contaminant database (244 sequences, 127,304 residues). Search parameters included the inferys_3.0.0_fragmentation prediction model with trypsin specificity and allowance for up to two missed cleavages. Peptide identification was limited to sequences of 7–30 amino acids, with up to three variable modifications and charge states from +1 to +6. A precursor mass tolerance of 20 ppm was used for database searching. After internal recalibration by CHIMERYS, precursor mass errors were recomputed for all reported peptide-spectrum matches (PSMs), yielding a median absolute error of 6.52 ppm (Figure S1). Variable modifications included TMT6/10/11 labeling at peptide N-termini and lysine residues, while cysteine carbamidomethylation was set as a static modification. Only PSMs and protein groups passing the 1% false discovery rate (FDR) threshold were used for downstream analyses.

Protein quantification was performed at the MS^2^ reporter ion level using the Reporter Ions Quantifier node in the Proteome Discoverer. The coisolation threshold was set to 50%, the normalized CHIMERYS coefficient threshold to 0.8, and the minimum average reporter ion signal-to-noise ratio to 10. Manufacturer-provided TMT isotope correction factors were applied during quantification. For protein-level quantification, both unique and razor peptides were used, with a minimum requirement of one unique peptide per protein. No additional normalization was applied across channels. To ensure rigorous reporting of quantified peptides and proteins, we classified the results into three categories: Quant, Pass, and Fail. Proteins and peptides were designated as Quant if they were master proteins, not flagged as contaminants, and contained at least one quantifiable signal across the reporter channels. Pass included all noncontaminant master proteins regardless of quantification, while fail encompassed contaminants and nonmaster proteins.

### Data Processing, Statistical Analysis, and Bioinformatics Analysis

For comparing the mean significance between methods, we conducted *t*-test in R within each three replicate samples for the benchmarking comparison between MiProChip and the manual control samples. The list of UniProt IDs of FDA-approved drug target proteins was obtained from The Human Protein Atlas^26^ (754 entities updated to 2025/09/10). The EGFR- and NSCLC-related pathway protein lists were constructed from the reference of the KEGG pathways.

The raw reporter ion intensities of PC9-H1975 results and MC38 results underwent log 2 transformation and two-step normalization in R: first, the median was subtracted for every column (sample-wise), and next the rows were centered to the mean for each group (in each plex of TMT). The coefficients of variations (CVs) of relative quantification were calculated after the normalization in R: CV = sqrt­(exp­((log­(SD) × log(2))^2^) – 1). For the evaluation of the Pearson correlation within each cell type, we used the function: cor­(mat, method = “pearson”, use = “pairwise.complete.obs”) to calculate all the possible and unique combinations between two TMT Channels’ intensity for the same type of cells. For MC38 single-cell data, we first remove the results from chip 1’s TMT 126 channel data, due to its significant low in reporter ion intensity. Next, the MC38 quantitative results after normalization are subjected for principal component analysis (PCA) in R by the prcomp function. Differential expression analysis was carried out using a custom R function. Proteins with fewer than three valid reporter ion intensity values per condition were excluded from statistical testing. Pairwise comparisons were performed between the control condition (MC38 untreated) and each treatment group (galectin-8, TGF-β, and combined galectin-8/TGF-β). For each protein, the log 2-fold change was defined as the difference between the mean log 2-transformed intensities of the treatment and reference groups. Statistical significance was evaluated using the two-sample *t* test, and *p*-values were adjusted for multiple testing using the Benjamini–Hochberg procedure to control the false discovery rate (FDR). Proteins with *q* < 0.05 and an absolute log 2-fold change ≥ log 2(1.2) were classified as significantly regulated, and the results are plotted as volcano plots via the ggplot2 function in R.

Differentially upregulated proteins (treated vs untreated) from each of the three treatment conditions were subjected to pathway enrichment analysis. Protein UniProt IDs were mapped in the STRING database,^27^ and enrichment was carried out against the Reactome Pathway Database^28^ using the *Homo sapiens* gene set as the background. Pathways with an adjusted *p*-value < 0.05 were considered significantly enriched. The top enriched pathways for each cell line were visualized to aid functional interpretation. For the sake of clarity, Reactome pathway terms were simplified according to the list provided (Table S2).

## Results and Discussion

### Design and Modular Architecture of MiProChip

The MiProChip introduces an integrated microfluidic workflow that addresses several limitations of conventional SCP platforms. By incorporating a tree-like design, the chip achieves higher throughput while maintaining distinct functional regions, including cell capture chambers, reaction vessels, and solid-phase extraction (SPE) columns for desalting (Figures 1A and S2). Within each reaction vessel, key sample processing steps, including cell lysis, protease digestion, TMT labeling and quenching, and acidification, are executed sequentially (Figure 1B). Each reagent is introduced only after completion of the preceding step, thereby minimizing cross-contamination and ensuring efficient, well-controlled reactions in the confined microreactor. Additionally, the integrated SPE columns streamline desalting, substantially reducing sample loss compared to conventional off-chip processing.

Each module of the MiProChip is further equipped with a 100-cell capturing unit to serve as a “boost” peptide carrier channel, a strategy demonstrated to enhance signal in previous off-chip SCP workflows.^29^ The single-cell chambers allow precise isolation of individual cells, while the boost peptide carriers enhance peptide signals by significantly enhancing MS1 signal intensity, thereby facilitating the detection of low amounts of proteins in single-cell samples (Figures 1C,D and S2). Following digestion and labeling in individual vessels, up to 12 single-cell samples along with one carrier sample are pooled and collectively flow through the activated desalting SPE column. While recent slide- or chip-based workflows have simplified pooling, they often rely on open-surface droplet pooling and droplet collection prior to LC injection. More generally, additional handling prior to LC injection can increase the risk of surface adsorption and contamination of low-input samples. By integrating the pooling step within the chip, MiProChip reduces the number of handling steps and improves the overall reliability of the TMT-proteomics workflow. Automated workflow control via pneumatic valves, operated through a custom graphical user interface in MATLAB software, further enables real-time workflow monitoring under a microscope. This allows the acquisition of single-cell images to be aligned with subsequent proteomic profiling (Figure 1E). Collectively, these features establish MiProChip as a robust and versatile solution for SCP, effectively bridging the gap between ultralow-input analysis and higher-throughput workflows.

### Efficient and Reproducible Single-Cell Capture

Cell capture performance was evaluated using non-small cell lung cancer PC9 cells at an input density of 250 cells/μL. Cell capture events were monitored in real time by bright-field microscopy, confirming both efficiency and reproducibility. Single-cell trapping was rapid and reliable, with most chambers capturing a cell within a few seconds (Movie S1). Capture efficiency was quantified by counting the number of cells passing through a chamber before a successful trapping event. For example, if the first cell was captured, efficiency was defined as 100%, whereas capturing a cell after two others had flowed through corresponded to 33%. Analysis of 36 chambers yielded an average capture efficiency of 53.1 ± 26.4%, representing an improvement over the previous chip (47.0 ± 25.0%)^19^ (Figure 2A). Regarding occupancy, 90% of the capture pillars contained exactly one cell, while only 10% contained two or three cells. This minor fraction of multicell captures is expected due to the stochastic nature of hydrodynamic trapping and can be excluded from downstream analysis if needed. On the other hand, the carrier chamber was designed to capture 100 cells. Each carrier chamber had a larger capture volume and incorporated an array of capture pillars to facilitate cell positioning. In repeated trials, the carrier chambers consistently captured ∼95–105 cells, and cell loading was completed within a few minutes, demonstrating that the carrier function did not significantly increase the workflow time.

**Figure 2:**
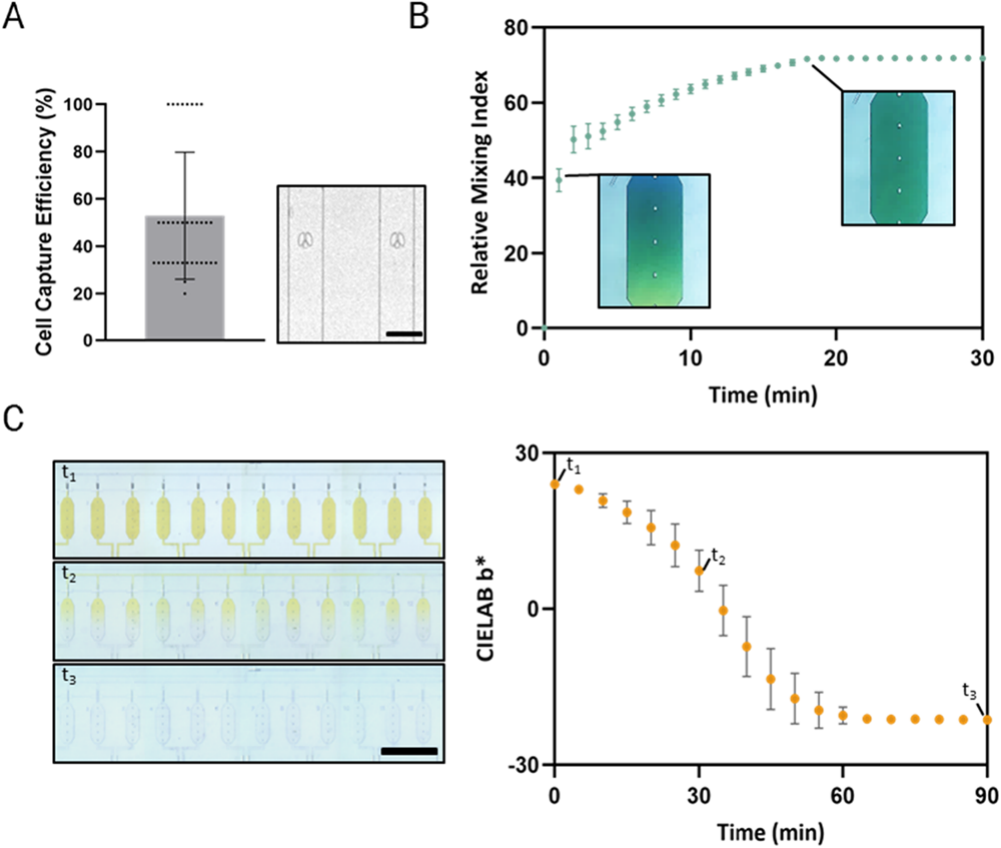
Functional characterizations of MiProChip. (A) Representative image (right) and the cell capture efficiency analysis (left) showing the efficient single-cell capturing of MiProChip. Note that each black dot shown in the efficiency analysis represents individual single-cell capture (*N* = 36). Scale bar: 100 μm. (B) Evaluation of mixing efficiency in the reaction vessel by time-lapse imaging (inset images) and relative mixing index (RMI) analysis of dye-based mixing experiments. Data are presented as mean ± standard deviation (SD) from three independent experiments (*N* = 3). (C) Time-lapse images (left) and corresponding preferential flow analysis (right) showing a MiProChip with a packed SPE column can be operated without concerning preferential flow. Data are presented as the chamber-to-chamber mean ± SD of the CIELAB b* value across 12 chambers at each time point (*N* = 12 chambers per time point). Scale bar: 3 mm.

In addition to capture efficiency, reproducibility was evaluated across multiple experiments using different chips. Performance metrics, including the time required for capture, the number of cells trapped, and the stability of capture efficiency, were consistent. These results indicate the robustness of the MiProChip design and its ability to reliably isolate cells in repeated experiments. Taken together, these findings demonstrate that the MiProChip achieves both high capture efficiency and reproducibility, providing a solid foundation for downstream proteomic processing.

### Rapid Reagent Mixing in Confined Nanoliter Chambers

Mixing efficiency within reaction chambers is a critical determinant for efficient proteomic workflows. At nanoliter volumes, diffusion alone is expected to be insufficient to homogenize reagents within a limited time window. This is particularly true for the multistep TMT labeling proteomics workflows, which include protein lysis, reduction, alkylation, digestion, and isotopic labeling. Uneven mixing in these steps can lead to incomplete reactions, variability between chambers, and ultimately a reduction in reproducibility and sensitivity. To address this challenge, MiProChip incorporates elongated octagonal chambers, a geometry that is expected to enhance convective flow and reduce stagnant regions by promoting internal recirculation.

Mixing performance of MiProChip was carefully evaluated using color-dye-based experiments by quantitative analysis of the relative mixing index (RMI).^30^ Under plate shaker-assisted agitation, the time to reach 50% of the total mixing (*t*_50_) was within 1 min, and complete mixing was typically observed within 20 min (Figures 2B and S3). Using the same *t*_50_ metric, our previous circular chamber required ∼2.5 min.^19^ These results indicate that the elongated octagonal geometry provides a slightly improved mixing efficiency compared with that of the earlier circular chamber. To ensure reactions can be mixed and reacted thoroughly, reagents used in the MiProChip workflow are incubated for at least 30 min. Meanwhile, the consistency of RMI values across chambers, as indicated by the standard deviation, demonstrated that the improved chamber geometry and mixing by the shaker were sufficient to overcome stochastic flow differences. This level of uniformity is essential for the proposed multiplexed workflows, where variability in one chamber could compromise the entire data set.

In summary, the elongated octagonal chambers in MiProChip provide rapid and uniform mixing, ensuring that biochemical reactions proceed under consistent conditions. This capability directly improves the reliability and reproducibility of downstream proteomic processing, making the device particularly suitable for high-throughput, single-cell applications.

### Eliminating Preferential Flow for Stable and Uniform Distribution

Flow stability is essential for a microfluidic operation. In devices with multiple parallel chambers, slight differences in channel geometry or resistance can cause preferential flow, where certain pathways receive disproportionately higher flow rates and thus, allowing more reagent to flow through. This effect will compromise uniformity across chambers, particularly during critical steps, such as labeling and desalting.

In the MiProChip, preferential flow was assessed by using dye-based experiments with either empty or bead-packed SPE columns. When columns were empty, some chambers exhibited visibly faster dye propagation, indicating lower resistance along these paths (Figure S4A). To quantify dye dispersion objectively, images of the chambers were analyzed in the CIELAB color space, and the b* parameter (representing the blue–yellow axis, b* > 0 indicates a shift toward yellow and b* < 0 indicates a shift toward blue) was used to monitor changes in dye intensity over time.^31,32^ This analysis confirmed the visual observation that certain chambers showed accelerated color development, reflecting a preferential flow. This uneven distribution suggested that reagents could reach the SPE column earlier than others, potentially introducing variability into the workflow. When the SPE column was packed with C18 beads, in contrast, the flow resistance was effectively equalized across all channels. The porous bead matrix acted as a balancing structure, forcing the flow to distribute more evenly. This configuration is consistent with the actual experimental setting, in which the columns are packed before sample elution; therefore, preferential flow is not expected to affect the experiments (Figure S4B).

Quantitative pressure testing showed that flow initiation through the packed SPE column could be achieved at >9 psi. A systematic comparison over the 10–12 psi range was performed to optimize chamber-to-chamber flow uniformity (Figures 2C and S4C). Among the tested conditions (10, 11, and 12 psi), the results showed that 11 psi could provide the most uniform flow distribution and robust operation and was therefore adopted as the standard operating pressure. This setting provided sufficient resistance to guarantee consistent flow distribution without overpressurizing the device (Figure 2C). Repeated trials confirmed the stability of this configuration. Dye tests showed uniform propagation across all chambers, and subsequent TMT experiments confirmed reproducible recovery across the parallel columns. These results demonstrate that bead packing serves a dual purpose: not only does it provide an effective medium for peptide binding and elution but it also stabilizes hydrodynamic conditions by equalizing resistance across chambers. The elimination of preferential flow is critical for proteomics, where reproducibility across parallel samples is required. Without flow stability, variability between chambers could introduce artifacts into peptide quantification and compromise the reliability of the results. By incorporating bead-packed SPE columns and optimization of the operating pressure, the MiProChip effectively overcame this challenge.

### Integration of TMT Multiplexing into MiProChip

A key innovation of MiProChip lies in the compatibility of TMT multiplexed labeling in the PDMS microfluidic workflow, a challenge due to intrinsic chemical incompatibilities between PDMS and TMT protocols. By systematic evaluation of solvent volatility, complete protein digestion, minimizing surface adsorption, and hydroxylamine (HA)-induced PDMS degradation, MiProChip successfully integrated TMT labeling, digestion, and desalting for SCP. MiProChip is designed with three modules; each module accommodates 12 sampling channels together with a carrier channel, enabling up to 12 single cells to be processed per module. With a 90 min LC gradient method (∼16 injections/day), the upper-bound throughput is therefore ∼192 single cells/day (12 × 16). To enable streamlined processing, several challenges were identified and addressed as following.

First, PDMS is gas-permeable and incompatible with highly volatile solvents. Thus, the use of 100% acetonitrile, commonly employed as the carrier solvent for TMT reagents, caused rapid solvent loss in microchannels (Figure S5A). To mitigate solvent loss, we tested the ACN percentage during TMT labeling by using a bulk peptide sample labeled with TMT-10. The results show that reducing acetonitrile content not only minimized solvent evaporation in microchannels but also preserved labeling efficiency (Figure S5B). Using 10% acetonitrile in 100 mM TEAB achieved 98.6 ± 0.4% labeling efficiency across conditions.

Furthermore, we attempted to reduce the proportion of missed cleavages of tryptic peptides, which was likely caused by the presence of protease inhibitors that impair enzyme (trypsin) activity under confined one-pot conditions. By comparing the single-cell samples with 100-cell carriers that utilize lysis buffer with or without protease inhibitor, our results showed that removing protease inhibitors from the lysis buffer significantly improved digestion efficiency, increasing the percentage of fully cleaved peptides from 77.6 ± 5.6 to 89.4 ± 1.5% on average (Figure S6).

Conventional BSA coating reduced nonspecific adsorption on PDMS microfluidic chips but interfered with TMT labeling, as abundant lysine and N-termini in BSA would compete with peptide labeling, leading to lower labeling efficiency (64.9 ± 8.6%). By substituting BSA with an amine-free, MS-compatible detergent, *N*-dodecyl-β-d-maltoside (DDM), our results showed that the labeling efficiency was substantially increased to 91.7 ± 4.1% and yielded a 12.5-fold increase in the number of protein identifications (BSA: 81 ± 52 protein groups; DDM: 1017 ± 127 protein groups) (Figure 3B,C). Furthermore, when formic acid (FA) was combined for sample acidification, hydroxylamine (HA), conventionally used to quench TMT labeling reactions, was found to cause bubble formation and optical darkening in the PDMS microchannels (Figure S7). Though the exact mechanism is currently unclear, it is speculated to arise from acid-induced HA decomposition, which releases gas and disrupts the PDMS matrix. To mitigate this issue, we tested various solvents and found that Tris buffer as the quenching reagent effectively prevented PDMS degradation while maintaining efficient TMT labeling. Together, these optimizations established, for the first time, that PDMS-based microfluidic devices can support robust TMT labeling, digestion, and desalting for carrier-assisted SCP.

**Figure 3:**
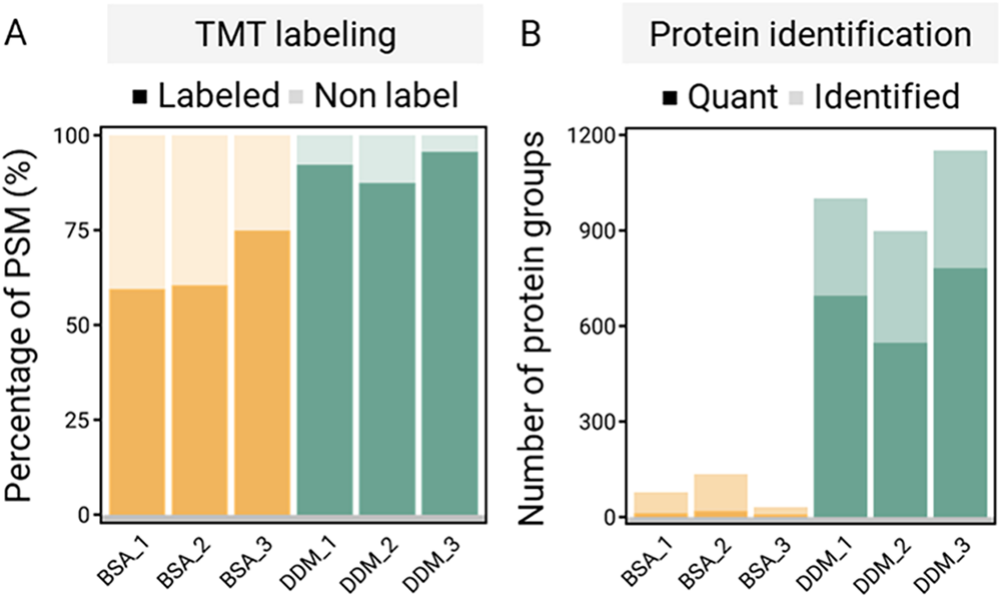
Optimization of cell lysis buffer and PDMS coating materials to improve SCP with TMT labeling in MiProChip. (A) TMT labeling efficiency increases using 0.01% (w/v) DDM (DDM) as PDMS coating materials compared to 0.01% (w/v) BSA coating (BSA). (B) Protein identification number also increases by replacing 0.01% (w/v) BSA with 0.01% (w/v) DDM.

### Analytical Performance of MiProChip

We then evaluated the analytical performance of MiProChip in triplicate experiments with PC9 and H1975 cells using a 100-cell carrier. The experiment was designed to accommodate 24 single cells on 3 modules within a chip; each module processes 8 single cells simultaneously (Scheme 1A), which significantly simplifies the traditional tube-based workflow (Scheme 1B).

We benchmarked the performance of MiProChip by two complementary controls: (1) limiting cell-dilution (manual aliquoting), which mimics conventional methods^33,34^ and (2) peptide dilution (bulk protein digests diluted to single-cell equivalents),^20^ providing an occupancy-independent benchmark for low-input samples. In a single module (one carrier channel and eight single-cell channels), MiProChip generated an average of 2775 ± 36 protein groups per run—3.6- and 1.7-fold higher than the limited cell-dilution (771 ± 78 protein groups) and peptide-dilution controls (1639 ± 198 protein groups), respectively. Similarly, MiProChip yielded 13,572 ± 574 peptides, representing 3.1- and 1.6-fold increases over the respective controls. Combining 3 modules from a MiProChip, a total of 3362 protein groups were identified from the 3 carrier channels and 24 single cells.

After optimization of TMT labeling (Figure 4B), MiProChip achieved high TMT labeling efficiency, with 91.3 ± 0.4% of PSMs carrying at least one TMT tag and 72.4 ± 1.2% being fully labeled. These values exceed those obtained from peptide-dilution controls (87.7 ± 1.0% ≥ 1 labeled PSM; 45.8 ± 2.2% fully labeled PSMs) and cell-dilution controls (74.4 ± 1.4% ≥ 1 labeled PSM; 36.5 ± 2.7% fully labeled PSMs). Proteolytic digestion efficiency in MiProChip remained high, with 88.9 ± 1.3% of PSMs without missed cleavage, comparable to that of peptide-dilution controls (88.4 ± 1.2%) (Table S3). Furthermore, manual cell-dilution controls contained a high percentage (48.3 ± 3.5%) of contaminant PSMs, whereas MiProChip generated negligible contamination (6.9 ± 0.6%). This disparity likely stems from manual handling and low peptide input in empty dilution channels. Conversely, MiProChip’s enclosed channel design minimizes sample loss and environmental exposure, enabling higher proteome coverage without contamination under single-cell input. Crucially, direct imaging confirms single-cell occupancy, eliminating dilution-based uncertainty and ensuring reliable proteomic interpretation.

**Figure 4:**
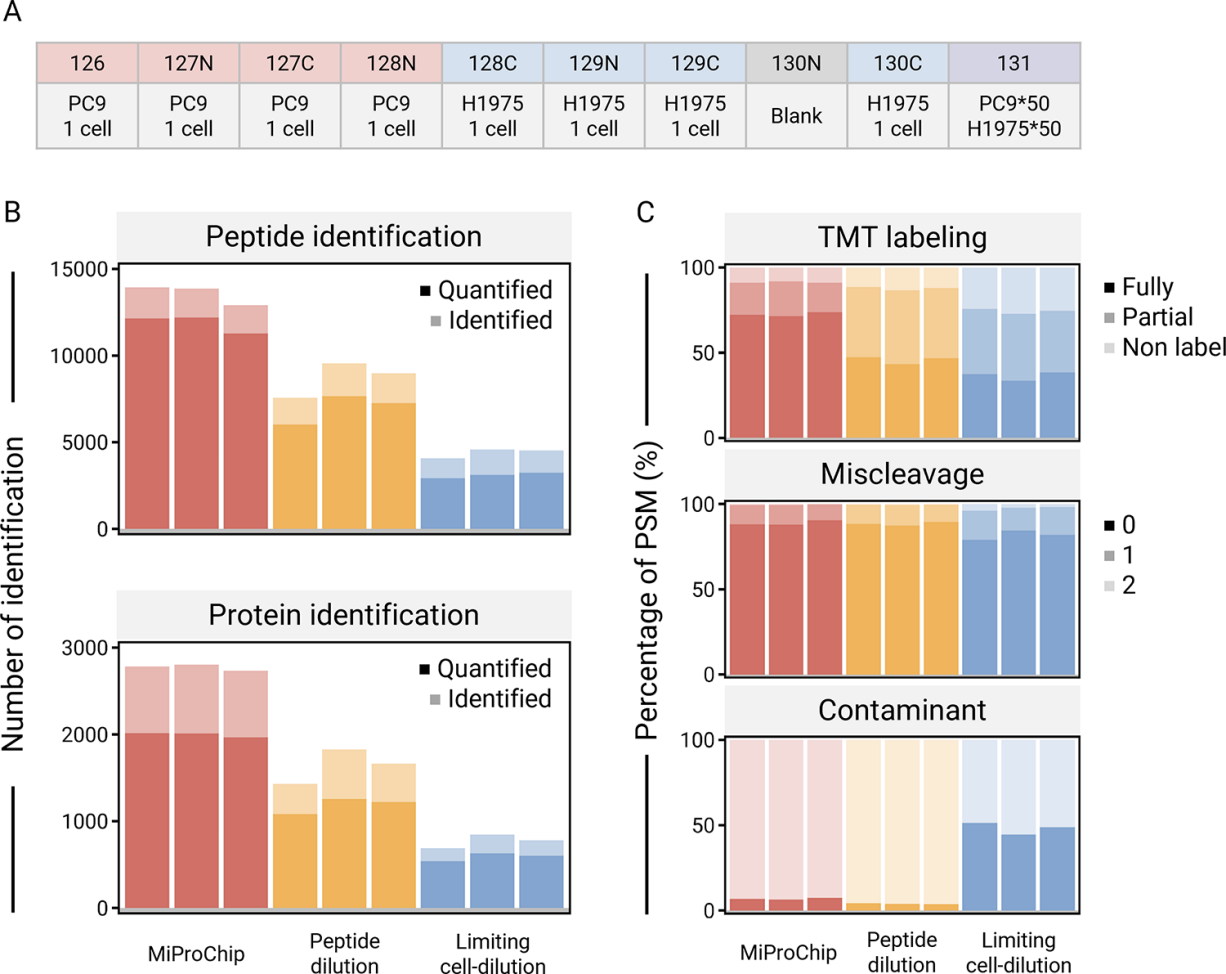
Comparison of MiProChip and manual processes for TMT-based SCP samples. The performance of MiProChip was compared to two manual processes: (1) Peptide-dilution: peptides were produced by diluting the sample amount to 0.2 and 20 ng equivalent to single-cell and hundred-cells channels, respectively, then manually combined after TMT labeling. (2) Limiting cell-dilution: cells were first diluted to one and a hundred cells for single-cell and hundred-cells channels, respectively, then manually combined after TMT labeling. (A) TMT channel layout in the MiProChip experiments. (B) Number of identified peptides and proteins of the three methods. (C) Comparison of proteomic profiling performance, including TMT labeling efficiency, peptide missed cleavages, and presence of contamination-related PSMs, obtained by the three methods.

Across 2775 ± 36 proteins identified in one module from MiProChip samples, an average of 1965 ± 23 proteins were quantified per single cell (Supporting Table S4). To evaluate technical robustness, we assessed protein “occupancy” across all single-cell channels. The results showed that 99.1 ± 2.3% of quantified proteins were detected in at least eight channels (Supporting Figure S8). Within the quantified proteins from three MiProChip modules, 73% (1462 proteins) are commonly quantified across the 3 modules (Figure 5A). These proteins possess similar dynamic ranges in protein abundance, spanning 2 orders of dynamic range of linear abundance (1.72–2.61 to 14.0–14.9 in log 2-transformed abundances) (Figure 5B). Among the quantifiable proteins, 73 proteins are FDA-approved drug targets, and 13 of them are clinically used or undergoing clinical trials in NSCLC treatment including PARP1, TOP1, ERK-2, HDAC1, RRM2, MEK1/2, EGFR, TOP2B, and PDK1. Notably, epidermal growth factor receptor (EGFR), a membrane protein, which is the primary drug target in the clinical routine, was identified and quantified at the single-cell level. Additionally, extracellular signal-regulated kinase 2 (ERK-2), a potential drug target, was also quantified with five unique peptides, such as ^345^ELIFEETAR^353^ for unambiguous identification by a long series of fragment *y*- and *b*-ions, and reporter ion spectra (Figure 5C). Furthermore, the quantification results showed carrier and single-cell channel ratios of 8.4 ± 3.3 on average (Figure S9). After normalization, the median CVs of protein abundance were 34, 31, and 30% for H1975 cells, PC9 cells, and carrier channels, respectively (Figure 5D). Spearman’s correlations within each cell line remained high (0.867 ± 0.008 to 0.896 ± 0.057; mean ± SD) regardless of the sample origin (Figure 5E), confirming the reproducibility of TMT reporter ion quantification by TMT labeling on MiProChip. Notably, after batch-effect correction, principal component analysis (PCA) distinctly separated PC9 and H1975 single cells, explaining 47% of the total variance (Figure S10).

**Figure 5:**
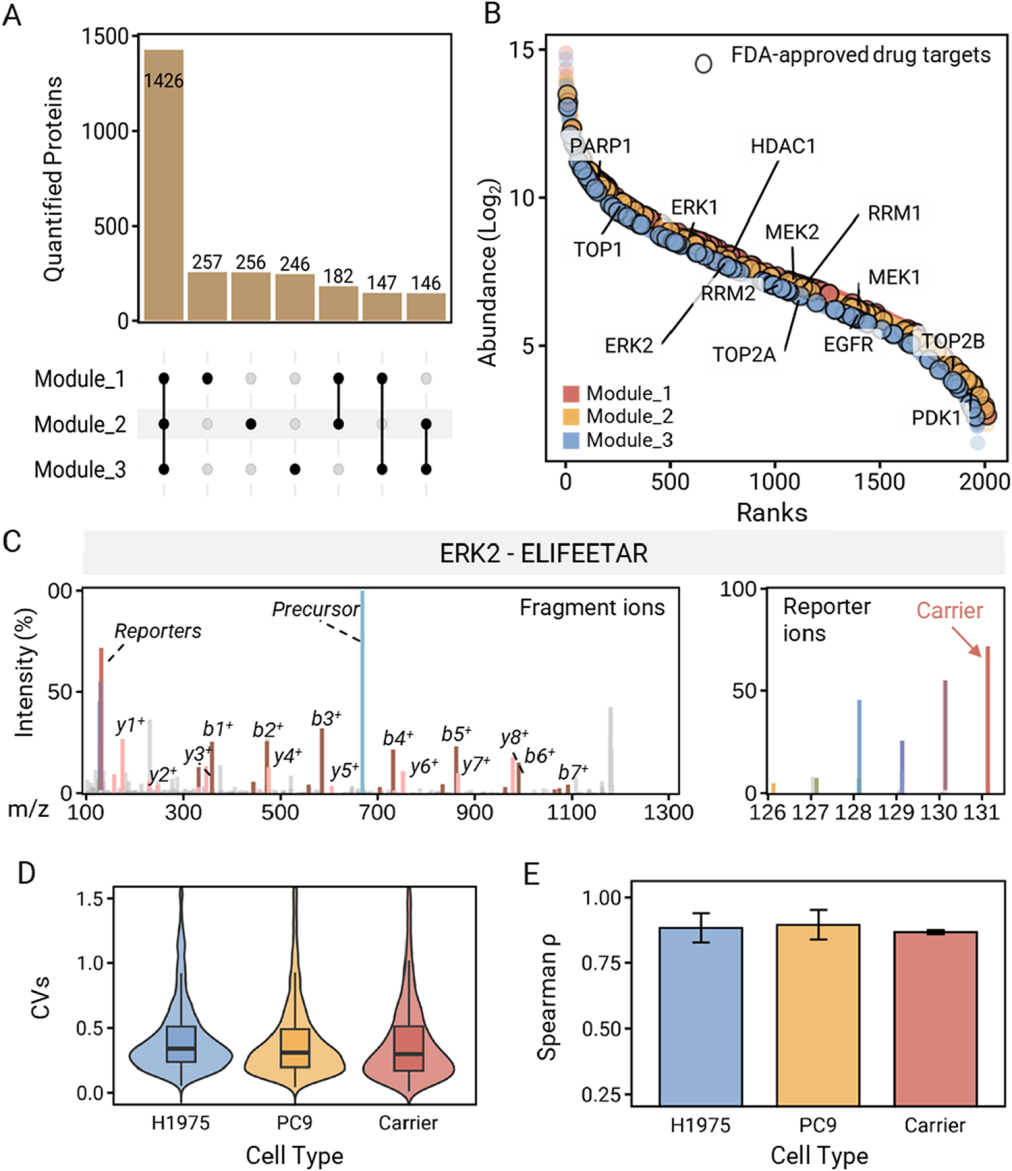
SCP analysis of PC9 and H1975 cells with MiProChip. (A) UpSet plot showcasing the overlapping of proteins quantified by three MiProChip modules (B) rank-abundance distribution of proteins from three replicate modules, the black circle represents FDA-approved drug targets. (C) Representative drug target ERK-2 peptide spectra with fragment and reporter ions. (D) Coefficient of variation of the normalized reporter ion intensity within each cell type H1975, PC9, and the pooled sample serves as the carrier sample. Violin shows kernel density; inner box spans Q1–Q3 with center line at the median; whiskers extend to the most extreme nonoutlier values (1.5 × interquartile range (IQR)). (E) Average Spearman’s correlations of all the possible combinations of normalized reporter ion intensity within each cell type H1975, PC9, and the carrier sample. Error bars represent mean ± SD from three independent experiments (*N* = 3).

Collectively, these results show that the optimized MiProChip achieves enhanced proteome coverage and reproducible quantification at the single-cell level, surpassing manual workflows. At such a low SCP level, the high sensitivity enabled the detection of FDA-approved drug protein targets.

### Application of MiProChip on Galectin-8 and TGF-β-Treated MC38 Colorectal Cancer (CRC) Cells

Having established the robustness and reproducibility of MiProChip using PC9 and H1975 cells, we applied the platform to investigate treatment-specific responses in MC38 cells cultured under four different conditions. We previously reported the antimetastatic role of galectin-8 in CRC cells, which is mediated through antagonizing the pro-metastatic effect of TGF-β.^35^ To demonstrate the capability of MiProChip for SCP profiling, we designed an experiment to assess treatment effects at the single-cell level. MC38 cells were cultured under four conditions: normal (control), treatment with recombinant galectin-8 (Gal-8), TGF-β, and combined recombinant proteins (Gal-8 + TGF-β). For the carrier channel, we pooled cells from all four conditions. In each MiProChip module, we analyzed 8 single cells alongside 100 mixed cells as carriers. By 2 MiProChips (5 modules in total), we profiled 40 single cells from the 4 treatment conditions (details in Table S1). Quantitative proteomics using bulk samples was also performed as a control.

Proteomic analysis quantified a total of 3199 proteins and quantified 1669 ± 261 proteins per single cell following normalization and batch-effect correction. Principal component analysis (PCA) showed separation between single-cell and carrier channels; two control cells were identified as outliers (Figure S11A,B). These two cells were excluded from subsequent analyses. PCA of the remaining cells showed separation between control and treated conditions; however, given the limited number of single-cell measurements per condition, this analysis is intended as a qualitative visualization of single-cell variability rather than as a statistically robust clustering assessment. Notably, the observed overlap between TGF-β and combined treatment conditions is consistent with the corresponding bulk proteomic analysis. In the PCA plot (Figure S12A), control cells are clearly separated from galectin-8, TGF-β, and combination treatment groups, indicating a pronounced treatment effect. The partial overlap between the galectin-8 and TGF-β groups suggests both shared and distinct functional roles. In contrast, the closer clustering of the TGF-β and combination groups implies a dominant contribution of TGF-β under the combined treatment conditions (Figure 6A).

**Figure 6:**
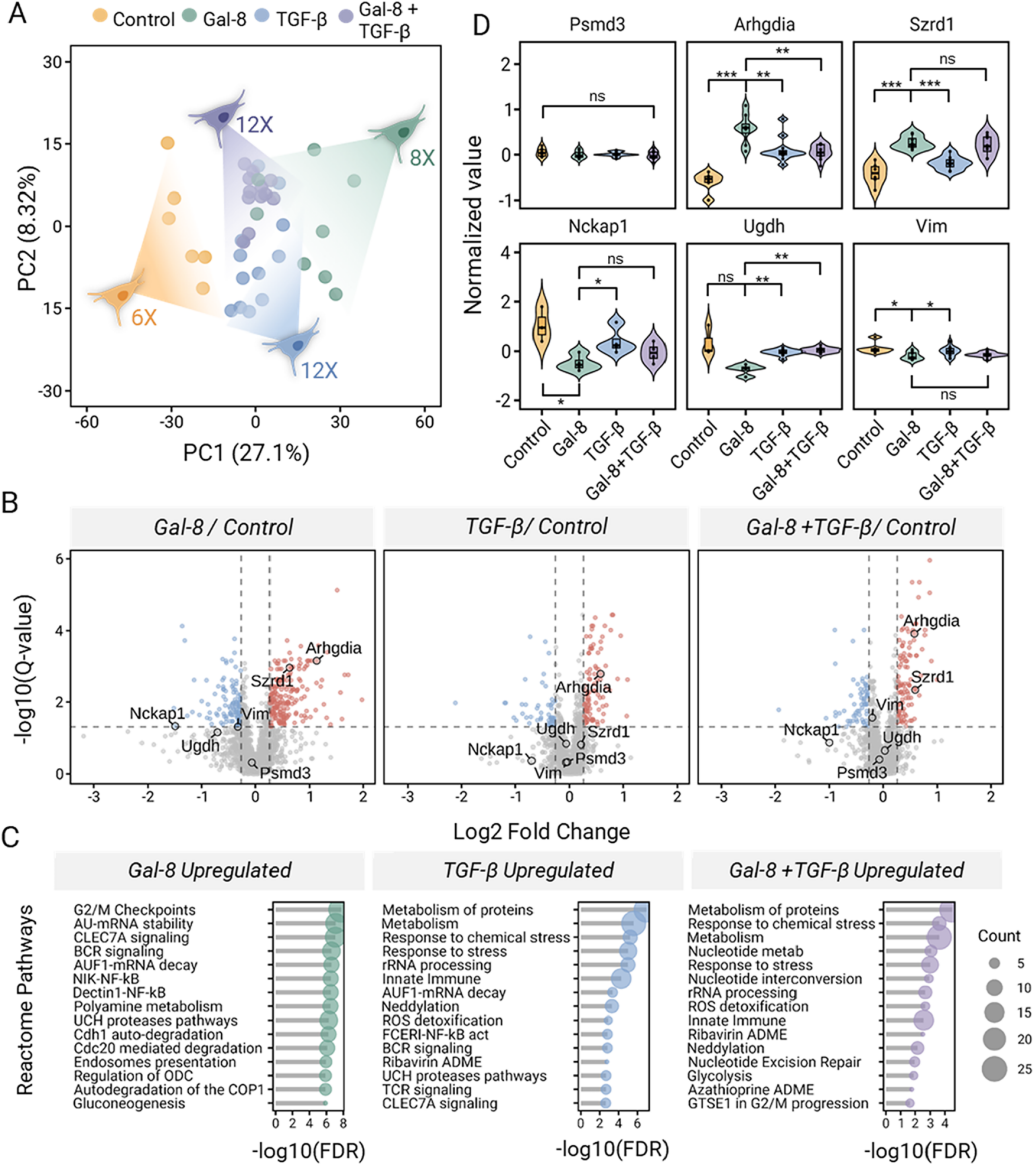
Application of MiProChip to the galectin-8-treated MC38 colorectal cancer cell line. (A) MC38 cells were subjected to galectin-8, TGF-β, or combined treatment. The principal component analysis of 38 single cells shows proteomic profile distribution across 4 conditions obtained by 2 MiProChips. (B) Volcano plot illustrating the differential protein expression between treatment and control cell lines. (C) Reactome pathway enrichment analysis of upregulated proteins in each comparison. (D) Normalized protein abundances of key representative proteins. Violin shows kernel density; inner box spans Q1–Q3 with the center line at the median; whiskers extend to the most extreme nonoutlier values (1.5 × IQR). Dots indicate individual cells.

To identify the regulatory or effector proteins associated with galectin-8 or TGF-β, a quantitative comparison was performed for differential expression analysis between control and treated cells. Compared with control cells, we identified 187, 94, and 102 upregulated proteins in galectin-8-treated cells, TGF-β-treated cells, and dual-treated cells, respectively (Figure 6B and Table S5). Similarly, 114, 48, and 63 downregulated proteins were associated with galectin-8, TGF-β and dual treatment, respectively. Pathway enrichment analysis (STRING, Reactome) revealed 144, 87, and 48 enriched pathways in the galectin-8, TGF-β, and dual-treatment groups, respectively. By selecting the top 25 pathways by STRING’s signals, we observed that 10 of them were commonly shared across all treatments, including pathways related to protein translation, RNA metabolism, and nonsense-mediated decay-related pathways. As a positive control, TGF-β-associated pathways were uniquely enriched in the TGF-β and dual-treatment groups such as FCERI-NF-κB, Neddylation, reactive oxygen species (ROS) detoxification, and glycolysis (Figure 6C). These pathways are known to regulate antiapoptosis, proliferation, and epithelial–mesenchymal transition (EMT). In contrast, cell cycle checkpoint pathways were enriched in the galectin-8 treatment group, suggesting a potential suppression of cell proliferation through enhanced regulatory mechanisms, such as in the G2/M checkpoint.

In addition to the cell population, SCP profiling allows exploration of the heterogeneity of protein expression levels across each single cell. We found that several metabolism, translation- and replication-related proteins are among the top 5% proteins and have stable expression levels with lowest CV across all cells (Figure S13). For example, 26S proteasome non-ATPase regulatory subunit 3 (PSMD3) remained unchanged in response to any treatment and showed consistent and stable expression across all of the cells (Figure 6D). For treatment-responsive proteins, such as Rho GDP-dissociation inhibitor 1 (ARHGDIA) and SUZ RNA Binding Domain Containing 1 (SZRD1), which are significantly elevated in galectin-8-treated cells, the protein abundance showed a wide range of elevated intensities. Importantly, these findings are consistent with their reported roles, with ARHGDIA promoting cell–cell adhesion and preventing the EMT process,^36^ and SZRD1 functions as a potential tumor suppressor in inhibiting cell proliferation.^37^ On the contrary, several proteins associated with metastasis, such as Nck-associated protein 1 (NCKAP1), UDP-glucose dehydrogenase (UGDH), and vimentin (Vim), were significantly downregulated in galectin-8-treated cells. NCKAP1 is part of the WAVE complex that regulates lamellipodia formation.^38^ UGDH has been found to be overexpressed and promotes metastasis in various cancers.^39,40^ Vimentin, a type III intermediate filament in mesenchymal cells, is one of the key regulatory markers of EMT.^41^ EMT is characterized by the downregulation of epithelial markers (e.g., E-cadherin) and upregulation of mesenchymal markers (e.g., vimentin), leading cells to proliferate, become invasive, and resist apoptosis.^42^ The exclusive downregulation of vimentin in galectin-8-treated and dual-treated cells (*p*-value < 0.05) suggested the antimetastasis potential of galectin-8 (Figure 6D and Table S6). This is partially consistent with our previous results when other CRC cell lines were used.^35^ Interestingly, this change in vimentin expression levels was not evident in the bulk DIA data (Figure S12B), reinforcing that the cellular drug treatment response could be masked in bulk proteomics measurement. Together, our results demonstrate that MiProChip can effectively resolve individual treatment-specific proteomic responses at the single-cell level, highlighting its potential for SCP screening and biological discovery from limited samples.

## Conclusions

In this study, we present MiProChip, a PDMS-based microfluidic platform that enables robust and high-throughput SCP. By incorporating tree-like flow channels, elongated octagonal reaction chambers, integrated carrier channels, bead-packed SPE columns, and on-chip pooling, MiProChip achieves efficient cell capture, rapid and uniform reagent mixing, and stable flow across parallel chambers. While TMT-based multiplexed quantification is long established in bulk proteomics, we adapted and optimized this chemistry for MiProChip, converting the traditionally manual workflow into a chip-compatible format that achieves high proteome coverage from single cells. The platform also resolves key challenges of on-chip digestion, TMT labeling, quenching, and desalting by addressing solvent incompatibility. Benchmarking against manually diluted controls showed that MiProChip improves proteome coverage, quantification reproducibility, and biochemical efficiency while minimizing sample loss and contamination. Applied to PC9, H1975, and MC38 cells, MiProChip identified thousands of proteins per cell and revealed treatment-specific proteomic alterations, demonstrating its potential as a sensitive and scalable SCP method for both basic and translational research.

## Supplementary Material







## Notes

Detailed description of biological information and experimental results; supporting methods; precursor mass accuracy across MiProChip replicates (Figure S1); design layout of the MiProChip (Figure S2); evaluation of the mixing efficiency within the reaction vessel of MiProChip by shaking (Figure S3); time-lapse images to examine the preferential flow in MiProChip (Figure S4); optimization of reagents in MiProChip operation (Figure S5); evaluation of protease inhibitors (PI) in the lysis buffer to affect missed cleavages in MiProChip SCP (Figure S6); effect of hydroxylamine (HA) and (formic) FA on the PDMS microchannels (Figure S7); single-cell channel occupancy of the three replicate MiProChip results (Figure S8); carrier-to-single-cell abundance ratio across MiProChip replicates (Figure S9); principal component analysis of MiProChip-derived proteomic profiles from H1975 and PC9 single cells (Figure S10); unnormalized quantification results of MC38 cells with different treatment groups (Figure S11); label-free DIA quantification of bulk MC38 cell proteomes (Figure S12); and pathway enrichment of the top 5% most stable proteins identified across 38 cells (Figure S13) (PDF)
Single-cell trapping with most chambers capturing a cell within a few seconds (AVI)
Experiment, chip, and TMT channel designs (Table S1); reactome pathway abbreviations (Table S2); PSMs, peptides, and proteins levels identification results of on-chip and manual processing single-cell samples (Table S3); single-cell channel wise protein quantification results (Table S4); differential expression analysis results for the treated MC38 cells (Table S5); and statistics of the key representative proteins (Table S6) (XLSX)

## ○

Department of Chemistry and Biochemistry, Brigham Young University, Provo, Utah 84602, United States

## ◆

Department of Biological Science and Technology, College of Engineering Bioscience, National Yang Ming Chiao Tung University, Hsinchu 300, Taiwan.

## References

[1] Ahmad R., Budnik B. (2023). A review of the current state of single-cell proteomics
and future perspective. Anal. Bioanal. Chem..

[2] Li S., Li S., Liu S., Ren Y. (2025). Mass Spectrometry-based Solutions
for Single-cell Proteomics. Genomics, Proteomics
Bioinf..

[3] Nalla L. V., Kanukolanu A., Yeduvaka M., Gajula S. N. R. (2025). Advancements
in Single-Cell Proteomics and Mass Spectrometry-Based Techniques for
Unmasking Cellular Diversity in Triple Negative Breast Cancer. Proteomics: Clin. Appl..

[4] Klein A. M., Mazutis L., Akartuna I., Tallapragada N., Veres A., Li V., Peshkin L., Weitz D. A., Kirschner M. W. (2015). Droplet barcoding for single-cell transcriptomics applied
to embryonic stem cells. Cell.

[5] Macosko E. Z., Basu A., Satija R., Nemesh J., Shekhar K., Goldman M., Tirosh I., Bialas A. R., Kamitaki N., Martersteck E. M. (2015). Highly Parallel Genome-wide Expression Profiling
of Individual Cells Using Nanoliter Droplets. Cell.

[6] Mereu E., Lafzi A., Moutinho C., Ziegenhain C., McCarthy D. J., Alvarez-Varela A., Batlle E., Sagar, Grun D., Lau J. K. (2020). Benchmarking single-cell RNA-sequencing protocols for cell atlas
projects. Nat. Biotechnol..

[7] Conte M. I., Fuentes-Trillo A., Dominguez Conde C. (2024). Opportunities and tradeoffs in single-cell
transcriptomic technologies. Trends Genet..

[8] Huang K., Xu Y., Feng T., Lan H., Ling F., Xiang H., Liu Q. (2024). The Advancement and Application of the Single-Cell Transcriptome
in Biological and Medical Research. Biology.

[9] Cui M., Cheng C., Zhang L. (2022). High-throughput proteomics: a methodological
mini-review. Lab. Invest..

[10] Gebreyesus S. T., Muneer G., Huang C.-C., Siyal A. A., Anand M., Chen Y.-J., Tu H.-L. (2023). Recent
advances in microfluidics
for single-cell functional proteomics. Lab Chip.

[11] Petrosius V., Schoof E. M. (2023). Recent
advances in the field of single-cell proteomics. Transl. Oncol..

[12] Wang D., Jin K., Ji J., Hu C., Du M., Belgaid Y., Shi S., Li J., Hu S., Nathan A. (2024). Active-matrix
digital microfluidics design for field programmable high-throughput
digitalized liquid handling. iScience.

[13] Shao X., Wang X., Guan S., Lin H., Yan G., Gao M., Deng C., Zhang X. (2018). Integrated Proteome Analysis Device
for Fast Single-Cell Protein Profiling. Anal.
Chem..

[14] Leipert J., Tholey A. (2019). Miniaturized sample preparation on
a digital microfluidics
device for sensitive bottom-up microproteomics of mammalian cells
using magnetic beads and mass spectrometry-compatible surfactants. Lab Chip.

[15] Kulak N. A., Pichler G., Paron I., Nagaraj N., Mann M. (2014). Minimal, encapsulated
proteomic-sample processing applied to copy-number estimation in eukaryotic
cells. Nat. Methods.

[16] Li Z. Y., Huang M., Wang X. K., Zhu Y., Li J. S., Wong C. C. L., Fang Q. (2018). Nanoliter-Scale Oil-Air-Droplet
Chip-Based
Single Cell Proteomic Analysis. Anal. Chem..

[17] Hughes C. S., Moggridge S., Muller T., Sorensen P. H., Morin G. B., Krijgsveld J. (2019). Single-pot, solid-phase-enhanced sample preparation
for proteomics experiments. Nat. Protoc..

[18] Zhu Y., Clair G., Chrisler W. B., Shen Y., Zhao R., Shukla A. K., Moore R. J., Misra R. S., Pryhuber G. S., Smith R. D. (2018). Proteomic Analysis of Single Mammalian Cells
Enabled by Microfluidic Nanodroplet Sample Preparation and Ultrasensitive
NanoLC-MS. Angew. Chem., Int. Ed..

[19] Gebreyesus S. T., Siyal A. A., Kitata R. B., Chen E. S.-W., Enkhbayar B., Angata T., Lin K.-I., Chen Y.-J., Tu H.-L. (2022). Streamlined
single-cell proteomics by an integrated microfluidic chip and data-independent
acquisition mass spectrometry. Nat. Commun..

[20] Budnik B., Levy E., Harmange G., Slavov N. (2018). SCoPE-MS: mass spectrometry
of single mammalian cells quantifies proteome heterogeneity during
cell differentiation. Genome Biol..

[21] Specht H., Emmott E., Petelski A. A., Huffman R. G., Perlman D. H., Serra M., Kharchenko P., Koller A., Slavov N. (2021). Single-cell
proteomic and transcriptomic analysis of macrophage heterogeneity
using SCoPE2. Genome Biol..

[22] Zhu Y., Piehowski P. D., Zhao R., Chen J., Shen Y., Moore R. J., Shukla A. K., Petyuk V. A., Campbell-Thompson M., Mathews C. E. (2018). Nanodroplet processing platform for deep and
quantitative proteome profiling of 10–100 mammalian cells. Nat. Commun..

[23] Ctortecka C., Hartlmayr D., Seth A., Mendjan S., Tourniaire G., Udeshi N. D., Carr S. A., Mechtler K. (2023). An Automated Nanowell-Array
Workflow for Quantitative Multiplexed Single-Cell Proteomics Sample
Preparation at High Sensitivity. Mol. Cell.
Proteomics.

[24] Frejno M., Berger M. T., Tüshaus J., Hogrebe A., Seefried F., Graber M., Samaras P., Ben Fredj S., Sukumar V., Eljagh L. (2025). Unifying
the analysis
of bottom-up proteomics data with CHIMERYS. Nat. Methods.

[25] Martin M. J., Orchard S., UniProt Consortium (2025). UniProt: the Universal
Protein Knowledgebase
in 2025. Nucleic Acids Res..

[26] Uhlén M., Fagerberg L., Hallström B. M., Lindskog C., Oksvold P., Mardinoglu A., Sivertsson Å., Kampf C., Sjöstedt E., Asplund A. (2015). Tissue-based map of the human proteome. Science.

[27] Szklarczyk D., Kirsch R., Koutrouli M., Nastou K., Mehryary F., Hachilif R., Gable A. L., Fang T., Doncheva N. T., Pyysalo S. (2023). The STRING database in 2023: protein-protein association
networks and functional enrichment analyses for any sequenced genome
of interest. Nucleic Acids Res..

[28] Milacic M., Beavers D., Conley P., Gong C., Gillespie M., Griss J., Haw R., Jassal B., Matthews L., May B. (2024). The Reactome Pathway Knowledgebase 2024. Nucleic Acids Res..

[29] Thompson A., Schafer J., Kuhn K., Kienle S., Schwarz J., Schmidt G., Neumann T., Hamon C. (2003). Tandem mass tags: a
novel quantification strategy for comparative analysis of complex
protein mixtures by MS/MS. Anal. Chem..

[30] An
Le N. H., Deng H., Devendran C., Akhtar N., Ma X., Pouton C., Chan H. K., Neild A., Alan T. (2020). Ultrafast star-shaped acoustic micromixer
for high throughput nanoparticle synthesis. Lab Chip.

[31] Komatsu T., Mohammadi S., Busa L. S., Maeki M., Ishida A., Tani H., Tokeshi M. (2016). Image analysis for a microfluidic
paper-based analytical device using the CIE L*a*b* color system. Analyst.

[32] Natsuhara D., Misawa S., Saito R., Shirai K., Okamoto S., Nagai M., Kitamura M., Shibata T. (2022). A microfluidic diagnostic
device with air plug-in valves for the simultaneous genetic detection
of various food allergens. Sci. Rep..

[33] Bernhard P., Feilen T., Rogg M., Fröhlich K., Cosenza-Contreras M., Hause F., Schell C., Schilling O. (2022). Proteome alterations
during clonal isolation of established human pancreatic cancer cell
lines. Cell. Mol. Life Sci..

[34] Eshghi A., Xie X., Hardie D., Chen M. X., Izaguirre F., Newman R., Zhu Y., Kelly R. T., Goodlett D. R. (2023). Sample
Preparation Methods for Targeted Single-Cell Proteomics. J. Proteome Res..

[35] Hsu T. H., Chang Y. C., Lee Y. Y., Chen C. L., Hsiao M., Lin F. R., Chen L. H., Lin C. H., Angata T., Liu F. T. (2024). B4GALT1-dependent
galectin-8 binding with TGF-beta
receptor suppresses colorectal cancer progression and metastasis. Cell Death Dis..

[36] Wibbe N., Steinbacher T., Tellkamp F., Beckmann N., Brinkmann F., Stecher M., Gerke V., Niessen C. M., Ebnet K. (2024). RhoGDI1 regulates
cell-cell junctions in polarized epithelial cells. Front. Cell Dev. Biol..

[37] Zhao N., Zhang G., He M., Huang H., Cao L., Yin A., Wang P., Wang L. (2017). SZRD1 is a Novel Protein that Functions
as a Potential Tumor Suppressor in Cervical Cancer. J. Cancer.

[38] Whitelaw J. A., Swaminathan K., Kage F., Machesky L. M. (2020). The WAVE Regulatory
Complex Is Required to Balance Protrusion and Adhesion in Migration. Cells.

[39] Harrington B. S., Kamdar R., Ning F., Korrapati S., Caminear M. W., Hernandez L. F., Butcher D., Edmondson E. F., Traficante N., Hendley J. (2023). UGDH promotes tumor-initiating
cells and a fibroinflammatory tumor microenvironment in ovarian cancer. J. Exp. Clin. Cancer Res..

[40] Fan M., Huo S., Guo Y., Wang R., Hao W., Zhang Z., Wang L., Zhao Y. (2024). UDP-glucose dehydrogenase
supports
autophagy-deficient PDAC growth via increasing hyaluronic acid biosynthesis. Cell Rep..

[41] Chen Z., Fang Z., Ma J. (2021). Regulatory mechanisms
and clinical
significance of vimentin in breast cancer. Biomed.
Pharmacother..

[42] Serrano-Gomez S. J., Maziveyi M., Alahari S. K. (2016). Regulation of epithelial-mesenchymal
transition through epigenetic and post-translational modifications. Mol. Cancer.

