# Supplementary material for: MiProChip: A Scalable Microfluidic Platform for Multiplexed Single-Cell Proteomics via Isobaric Labeling
Source: Anal Chem. 2026 Feb 25;98(9):6874–86. doi: 10.1021/acs.analchem.5c07275 (PMC12980487; doi:10.1021/acs.analchem.5c07275)
Supplement: Supplementary file 1 [file ac5c07275_si_001.pdf]

Supporting Information

MiProChip: A Scalable Microfluidic Platform for Multiplexed Single-cell Proteomics via Isobaric Labeling

Tsai-Fang Chou[1,†], Huan-Chi Chiu[1,2,†], Sofani Tafesse Gebreyesus[1,7], Guan-Fu Chen[1], Yi-Ju Chen[1,8], Abigail Ruth F. Velasquez[3,4,5], Kuo-I Lin[3,4], Yu-Ju Chen[1,2,], Hsiung-Lin Tu[1,6,*]*

[1]Institute of Chemistry, Academia Sinica, Taipei 11529, Taiwan.

[2]Department of Chemistry, National Taiwan University, Taipei 10617, Taiwan.

[3]Genomics Research Center, Academia Sinica, Taipei 115, Taiwan.

[4]Chemical Biology and Molecular Biophysics, Taiwan International Graduate Program, Academia Sinica, Taipei 115, Taiwan

[5]Graduate Institute of Biochemical Sciences, National Taiwan University, Taipei 110, Taiwan

[6]Genome and Systems Biology Degree Program, Academia Sinica and National Taiwan University, Taipei 110, Taiwan

[7]Current address: Department of Chemistry and Biochemistry, Brigham Young University, Provo 84602, Utah, USA

[8]Current address: Department of Biological Science and Technology, College of Engineering Bioscience, National Yang Ming Chiao Tung University, Hsinchu 300, Taiwan

*Correspondence: yujuchen@as.edu.tw (YJ Chen), hltu@gate.sinica.edu.tw (HL Tu)

†These two authors contributed equally.

Table of Contents

Supporting Methods S2

Labeling efficiency test for ACN concentration during TMT labeling S2

MiProChip reagent optimization experiments S2

Bulk MC38 DIA analysis S2

LC–MS/MS analysis and proteomic database searching S2

DIA analysis for bulk MC38 samples S3

Supporting Figures S4

Figure S1. Precursor mass accuracy across MiProChip replicates S4

Figure S2: Design layout of the MiProChip. S5

Figure S3. Evaluation of the mixing efficiency within the reaction vessel of MiProChip by shaking. S6

Figure S4. Time-lapse images to examine the preferential flow in MiProChip. S7

Figure S5. Optimization of reagents in MiProChip operation. S8

Figure S6. Evaluation of protease inhibitors (PI) in lysis buffer to affect missed cleavages in MiProChip SCP S9

Figure S7. The effect of Hydroxylamine (HA) and (Formic) FA on the PDMS microchannels. S10

Figure S8. The single cell channel occupancy of the triplicate MiProChip results S11

Figure S9. Carrier-to-single-cell abundance ratio across MiProChip replicates S12

Figure S10. Principal component analysis of MiProChip-derived proteomic profiles from H1975 and PC9 single cells. S13

Figure S11. Unnormalized quantification results of MC38 cells with different treatment groups S14

Figure S12. Label-free DIA quantification of bulk MC38 cell proteomes. S15

Figure S13. Pathway enrichment of the top 5% most stable proteins identified across 38 cells. S16

Supporting Methods

Labeling efficiency test for ACN concentration during TMT labeling

Peptides generated from PC9 cell lysates were labeled with TMT0 while varying acetonitrile (ACN) content during (i) TMT reagent dissolution and (ii) the labeling reaction. TMT0 reagents were dissolved in 100%, 50%, or 30% ACN, and labeling reactions were performed at final ACN concentrations of either 30% or 10%. Labeling proceeded for 1 h and was quenched with hydroxylamine. Samples were desalted using StageTips and analyzed in DDA mode on an Orbitrap Fusion Lumos.

MiProChip reagent optimization experiments

MiProChip optimization experiments evaluating protease inhibitor supplementation and chip surface passivation were performed using six single-cell channels and a 100-cell carrier. The TMT channel layout was: 126 (carrier) and 128C–131 (single-cell channels). Samples were prepared using the same workflow described in the Methods section, except that a protease inhibitor cocktail was added to the RapiGest lysis buffer (final 1×). For surface passivation, MiProChip channels were coated with 0.01% DDM for 1 h, rinsed with PBS, and dried under nitrogen. For BSA versus DDM comparisons, the same coating procedure was used, except DDM was replaced with BSA. Samples were analyzed in DDA mode on an Orbitrap Fusion Lumos using a 90-min LC gradient.

Bulk MC38 DIA analysis

MC38 cells from four conditions were collected in three biological replicates, washed with PBS, and lysed using an SDC-based lysis buffer. From each lysate, 50 µg protein was digested overnight with Lys-C/trypsin and desalted using SDB-XC-C18 StageTips. Desalted peptides were dried by SpeedVac at 45 °C, reconstituted in 0.1% formic acid (FA), and prepared for LC–MS analysis.

LC–MS/MS analysis and proteomic database searching

All optimization experiment samples were analyzed on an Orbitrap Fusion Lumos coupled to an UltiMate 3000 RSLCnano system. Desalted peptides were resuspended in 0.1% FA, and 4.5 µL was injected. Peptides were separated using a 90-min gradient and acquired in DDA mode using the same LC–MS/MS settings described in the main Methods (LC–MS/MS Analysis). Raw files were searched using Proteome Discoverer v2.5 (Thermo Fisher Scientific). Peptide identification was restricted to 7–30 amino acids, allowing up to three variable modifications per peptide and charge states from +1 to +6. Precursor mass tolerance was set to 10 ppm. Variable modifications included TMT6 or TMT0 labeling on peptide N-termini and lysine residues, oxidation on methionine residues and deamidation on asparagine and glutamine residues, and cysteine

carbamidomethylation was set as a fixed modification. PSMs were filtered to 1% FDR.

DIA analysis for bulk MC38 samples

Bulk MC38 peptides were analyzed on an Orbitrap Fusion Lumos coupled to an UltiMate 3000 RSLCnano. Peptides were resuspended to 100 ng/µL and 1 µL was injected. LC separation used a 60-min gradient: 2% B at 0 min; 10% B at 3 min; linear to 15% B at 40 min; 25% B at 48 min; 45% B at 53 min; and 90% B at 60 min.

DIA acquisition used full MS scans at 120,000 resolution (AGC 100%, maximum injection time 50 ms) over 340–1250 m/z. MS/MS scans were acquired using a 15 m/z isolation window, sequentially covering 350–800 m/z, with HCD (NCE 25%). Fragment spectra were acquired in the Orbitrap at 15,000 resolution (AGC 200%, maximum injection time 22 ms). DIA raw files were processed in Spectronaut v1.9 using directDIA with trypsin cleavage rules and default settings.

Supporting Figures

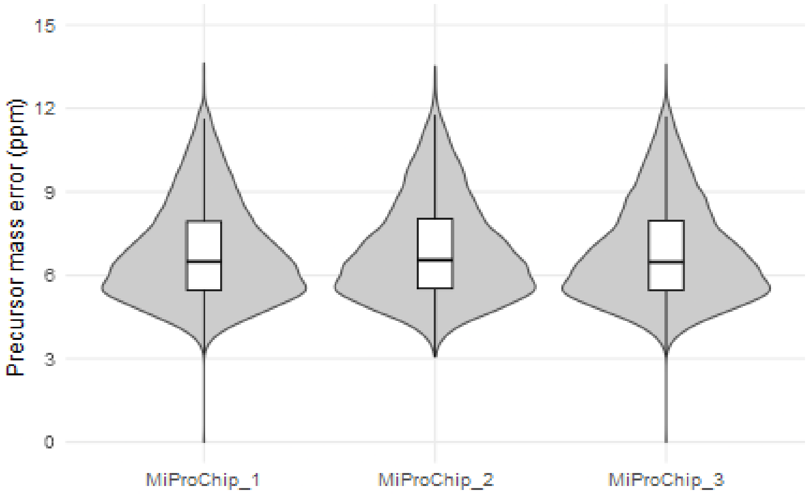

Figure S1. Precursor mass accuracy across MiProChip replicates.

Violin plots show the distribution of precursor mass errors (ppm) for all reported PSMs in three replicate MiProChip runs (MiProChip_1–3). Boxplots indicate the median and interquartile range.

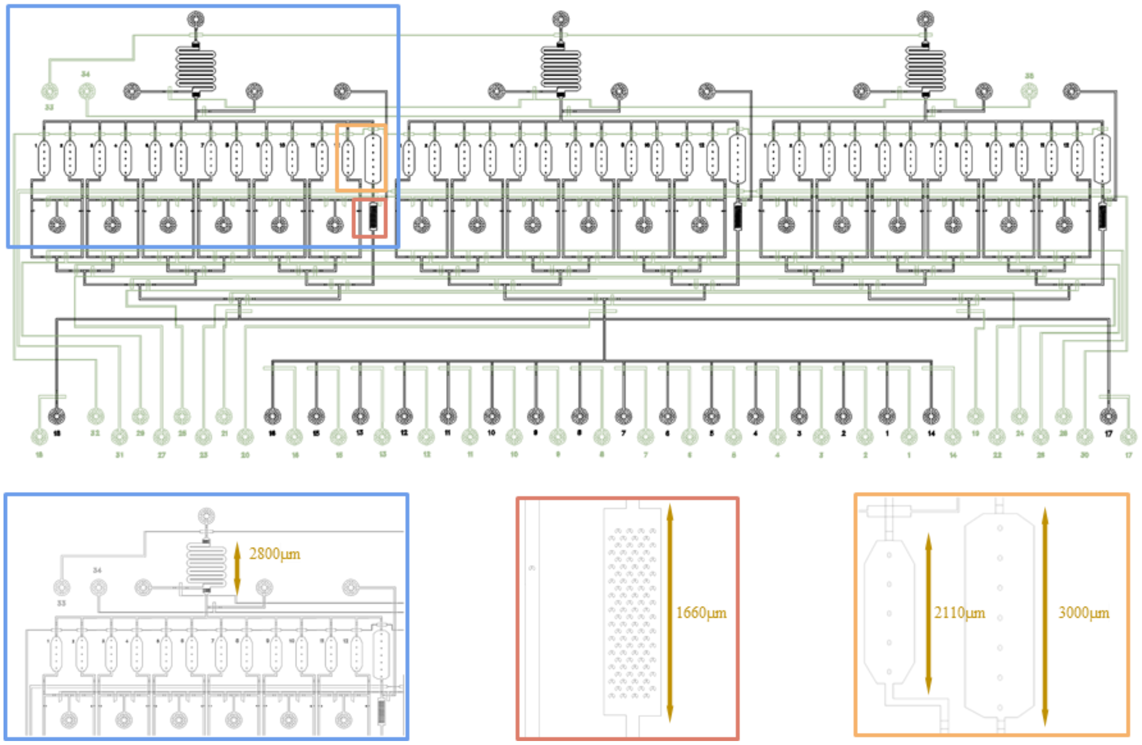

Figure S2: Design layout of the MiProChip.

The lower panels provide magnified views highlighting structural details and dimensions of the MiProChip.

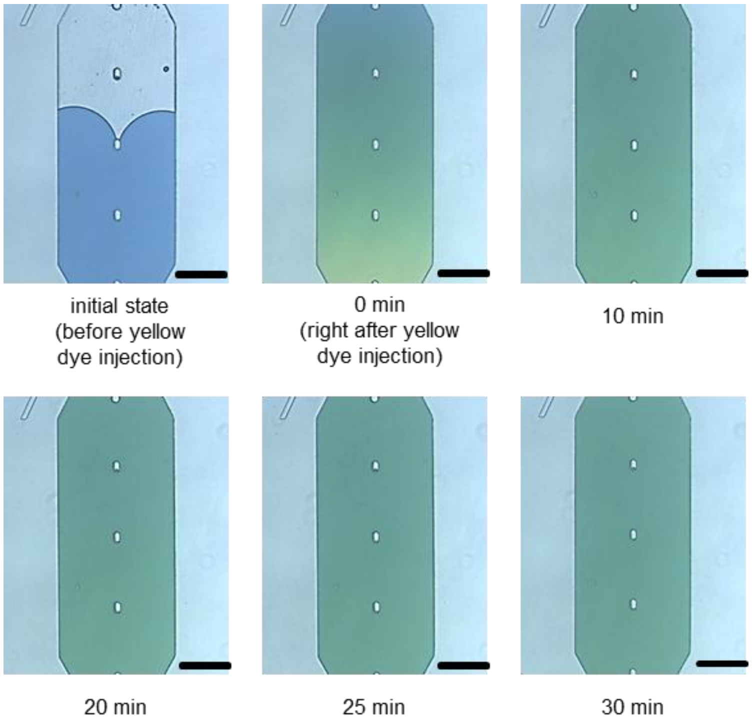

Figure S3. Evaluation of the mixing efficiency within the reaction vessel of MiProChip by shaking.

Time-lapse images of a reaction vessel during the dye-mixing experiment. Scale bar: 350 µm.

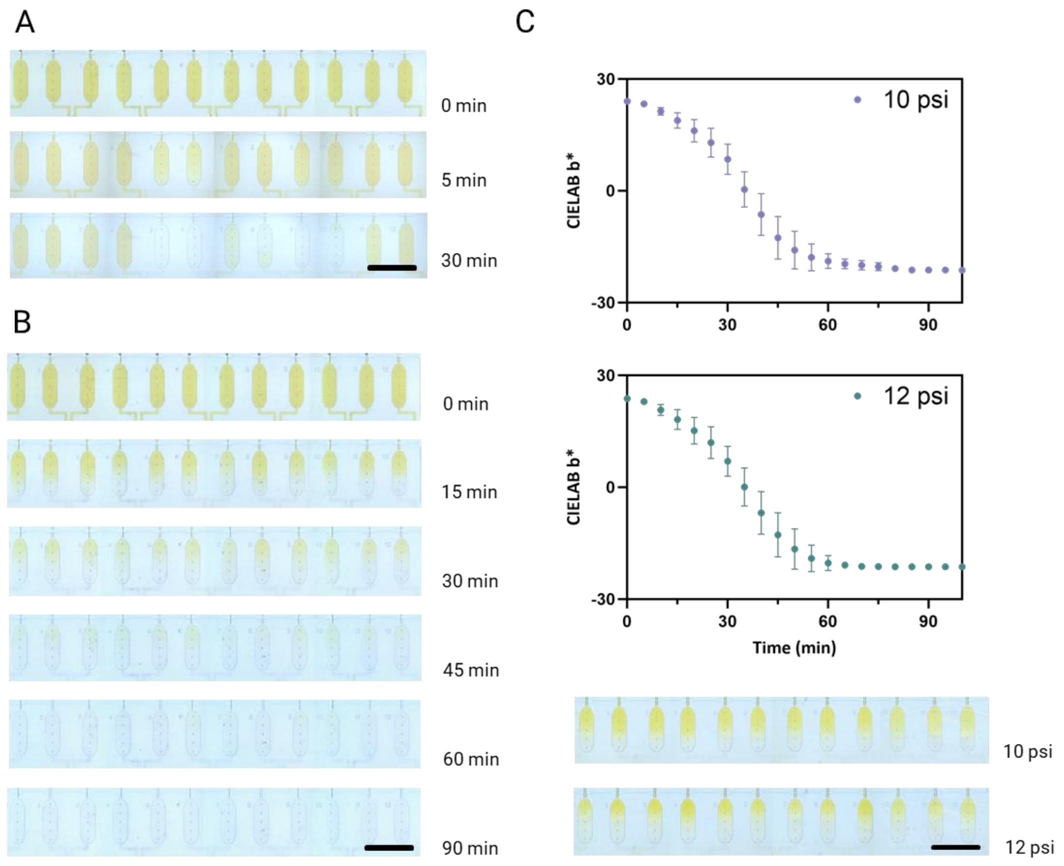

Figure S4. Time-lapse images to examine the preferential flow in MiProChip.

An image of all reaction vessels during buffer exchange at 11 psi with (A) empty SPE column and (B) C18 beads-packed SPE column. Scale bar: 2 mm. (C) The preferential flow analysis at 10 and 12 psi (top) and representative time-lapse images at 15-min for each pressure (bottom). Data are presented as the chamber-to-chamber mean ± SD of the CIELAB b* value across 12 chambers at each time point (N = 12 chambers per time point). Scale bar: 2 mm.

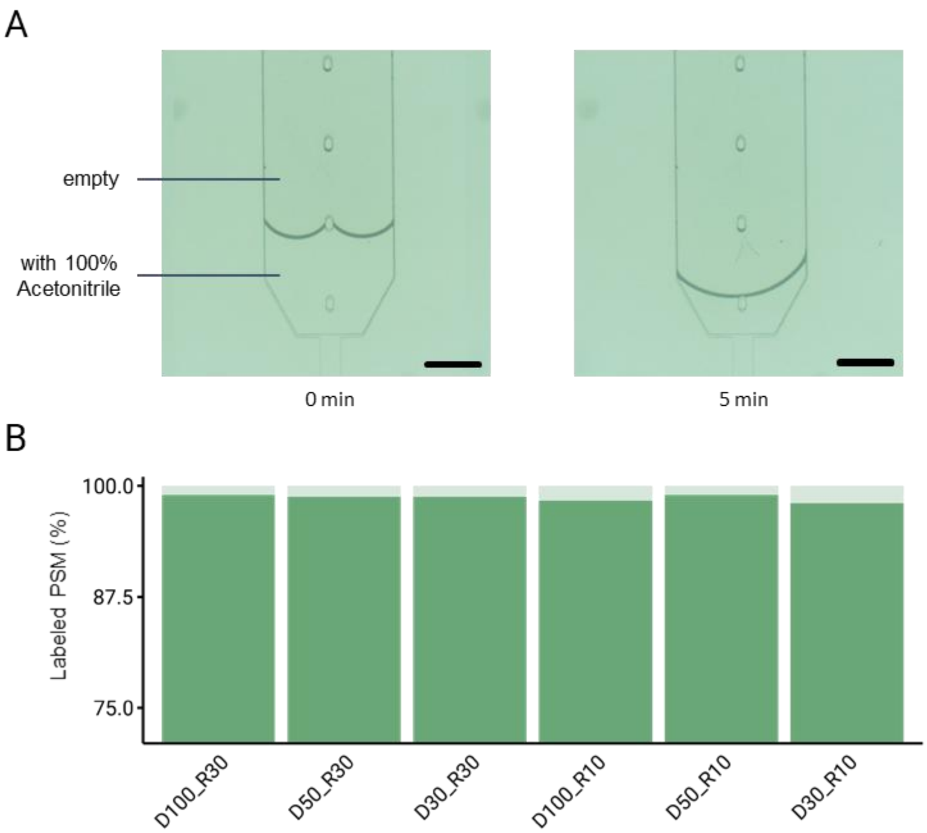

Figure S5. Optimization of reagents in MiProChip operation.

(A) Substantial evaporation of Acetonitrile (100%) in MiProChip. Approximately 20 nL of ACN was lost within 5 min. Scale bar: 350 µm. (B) Systematic evaluation of TMT-labeled PSMs percentages under various reaction conditions to identify the optimal chip-compatible carrier solvent. For instance, D100_R30 means dissolving TMT in 100% ACN, then performing peptide labeling reactions in 30% ACN.

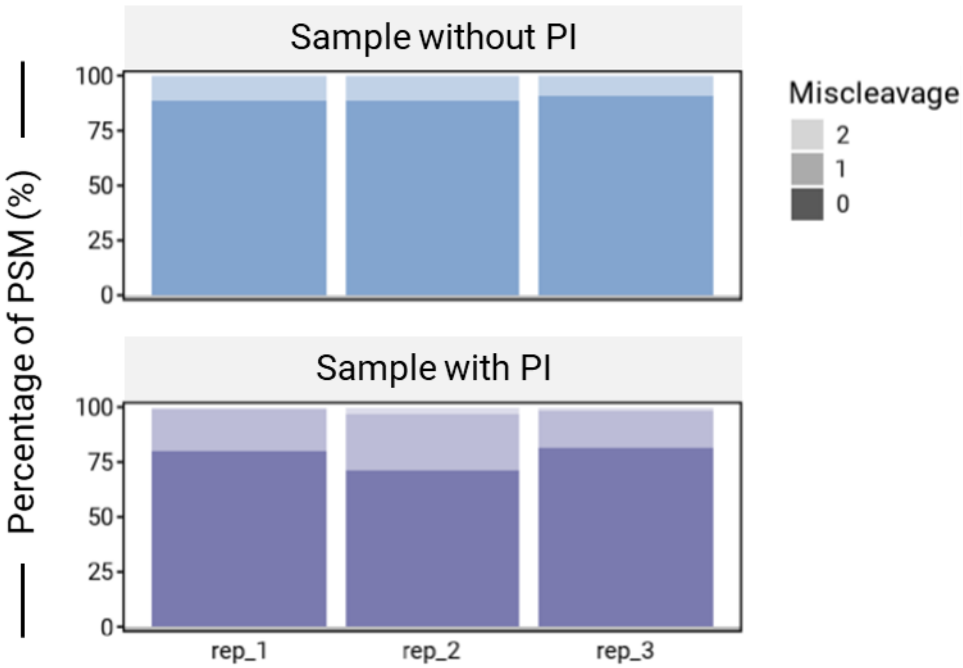

Figure S6. Evaluation of protease inhibitors (PI) in lysis buffer to affect missed cleavages in MiProChip SCP.

Stacked bars show the percentage of PSMs with 0, 1, or 2 missed cleavages across three replicates for samples prepared without (top) or with PI (bottom). The presence of PI shows a higher fraction of missed cleavage of PSMs (22.4%) than the condition without PI (10.6%). It is noted that these data were acquired on different instruments (Orbitrap Eclipse vs. Orbitrap Lumos) and from different cell samples.

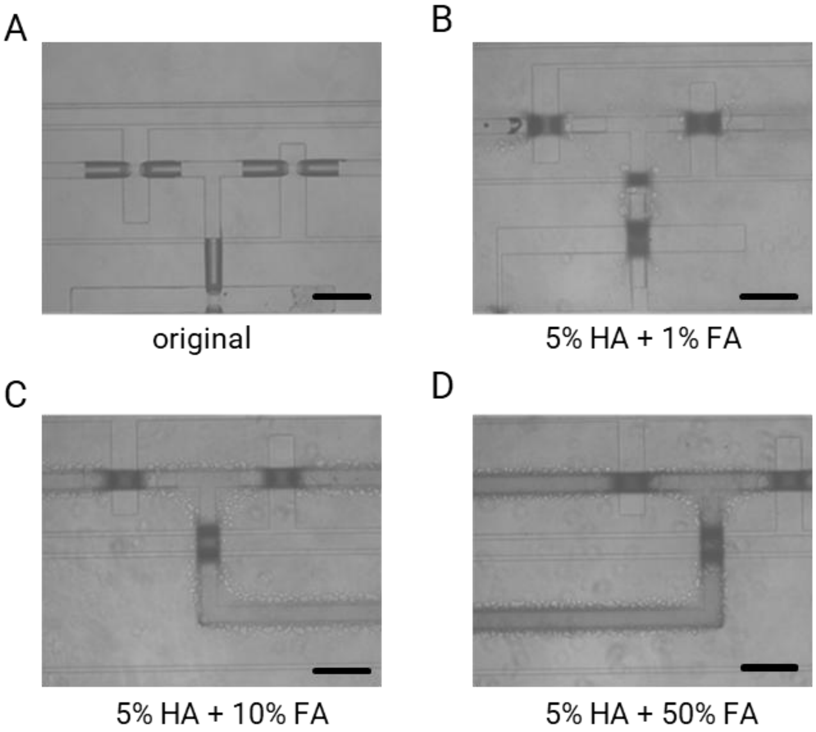

Figure S7. The effect of Hydroxylamine (HA) and (Formic) FA on the PDMS microchannels. Representative bright-field images of (A) original microchannel (without HA and FA) and (B)-(D) those treated with different percentages of HA and FA as indicated in the figures. Scale bar: 350 μm.

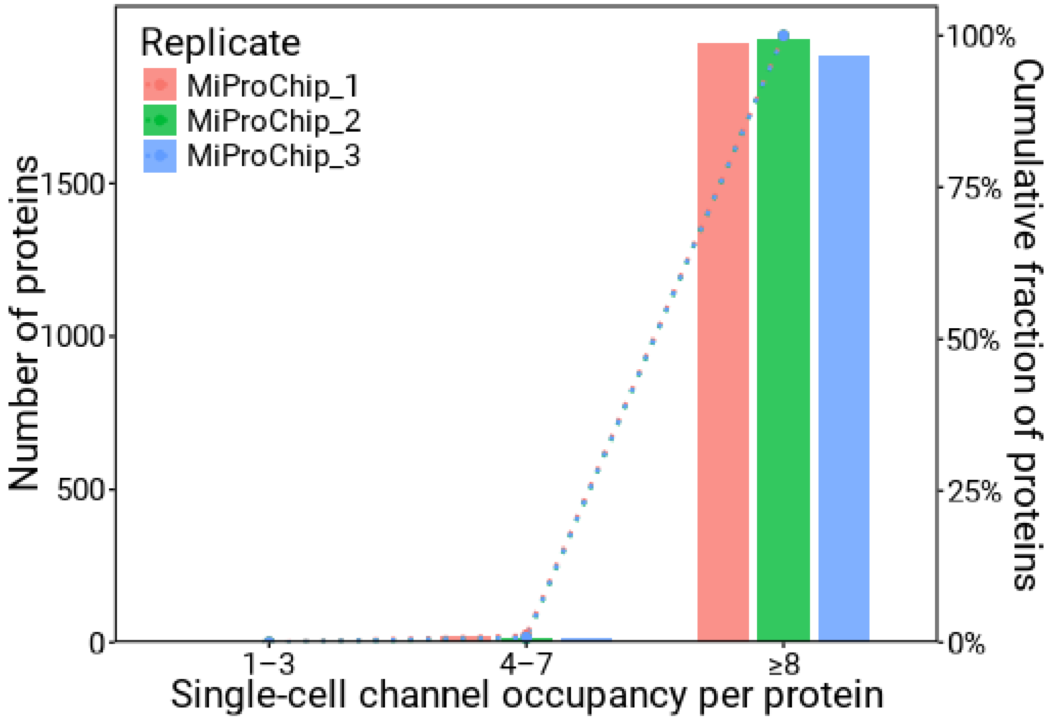

Figure S8. The single cell channel occupancy of the triplicate MiProChip results.

Bar plots show the number of quantified proteins binned by the number of single-cell channels in which they were quantified (1–3, 4–7, or ≥8) for three replicate MiProChip runs (MiProChip_1–3; left y-axis). Dotted lines indicate the cumulative fraction of quantified proteins (right y-axis). Across replicates, 99.1 ± 2.3% of quantified proteins were quantified in ≥8 single-cell channels (i.e., all single-cell channels).

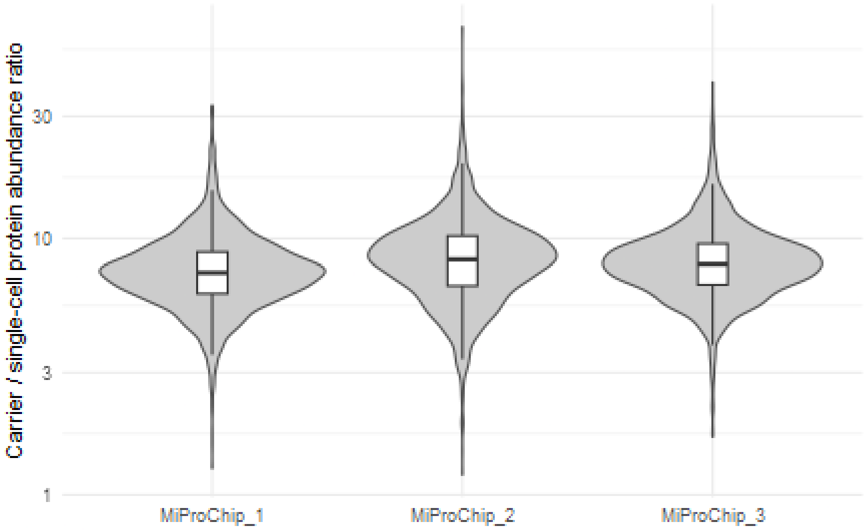

Figure S9. Carrier-to-single-cell abundance ratio across MiProChip replicates.
Violin plots show the distribution of carrier and single-cell protein abundance ratios for quantified proteins in three replicate MiProChip runs (MiProChip_1–3). Boxplots indicate the median and interquartile range.

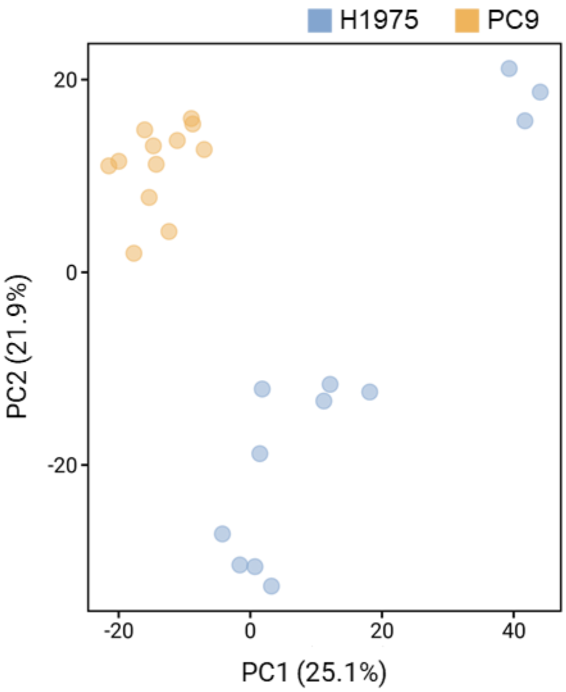

Figure S10. Principal component analysis of MiProChip-derived proteomic profiles from H1975 and PC9 single cells.

The result shows that utilizing MiProChip we can capture the differential expression in proteomics of two distinct cell lines.

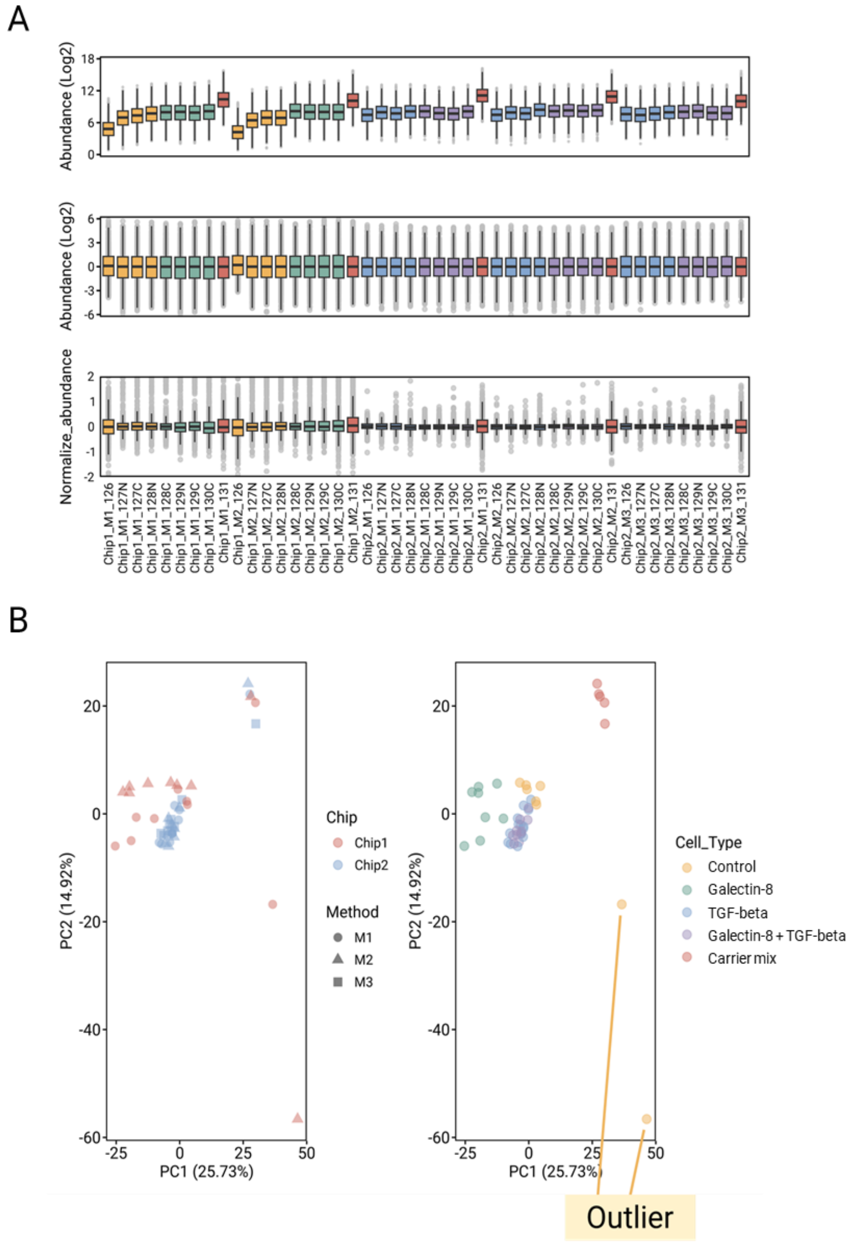

Figure S11. Unnormalized quantification results of MC38 cells with different treatment groups.

(A) The raw Log2 abundance, column-based normalization, and grouped row-based batch effect correction of the 24 cells. (B) The PCA results for the 40 single cells and the mixed carrier samples.

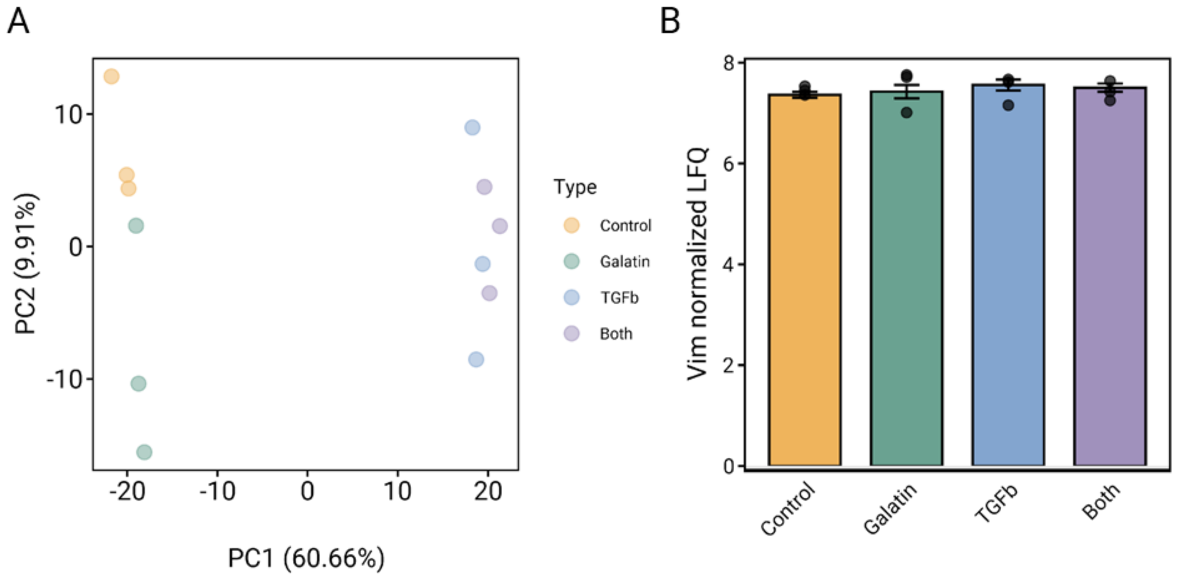

Figure S12. Label-free DIA quantification of bulk MC38 cell proteomes.

(A) PCA of bulk protein abundance profiles. (B) Vimentin LFQ intensities across the four treatment conditions.

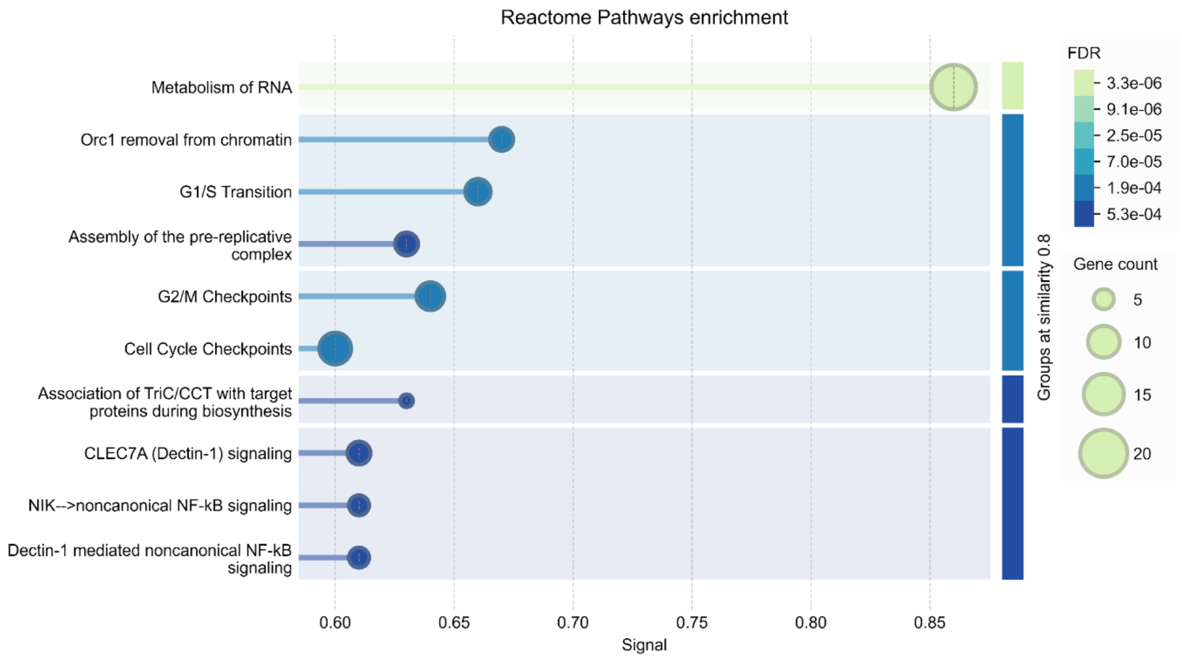

Figure S13. Pathway enrichment of the top 5% most stable proteins identified across 38 cells. We performed Reactome pathway enrichment using the STRING online platform on the proteins with the lowest 5% CVs among all commonly quantified proteins across 38 single cells subjected to four treatment conditions (Control, Galectin-8, TGF-β, and Galectin-8 + TGF-β).
